# Characterization of a fungal competition factor: Production of a conidial cell-wall associated antifungal peptide

**DOI:** 10.1371/journal.ppat.1008518

**Published:** 2020-04-23

**Authors:** Sheng Tong, Maolian Li, Nemat O. Keyhani, Yu Liu, Min Yuan, Dongmei Lin, Dan Jin, Xianbi Li, Yan Pei, Yanhua Fan

**Affiliations:** 1 Biotechnology Research Center, Southwest University, Chongqing, P.R. China; 2 Department of Microbiology and Cell Science, Institute of Food and Agricultural Sciences, University of Florida, Gainesville, Florida, United States of America; 3 College of Biotechnology, Southwest University, Chongqing, P. R. China; 4 Chongqing Key Laboratory of Plant Resource Conservation and Germplasm Innovation, Southwest University, Chongqing, P.R. China; University of Wisconsin-Madison, UNITED STATES

## Abstract

Competition is one of the fundamental driving forces of natural selection. *Beauveria bassiana* is a soil and plant phylloplane/root fungus capable of parasitizing insect hosts. Soil and plant environments are often enriched with other fungi against which *B*. *bassiana* competes for survival. Here, we report an antifungal peptide (BbAFP1), specifically expressed and localized to the conidial cell wall and is released into the surrounding microenvironment inhibiting growth of competing fungi. *B*. *bassiana* strains expressing BbAFP1, including overexpression strains, inhibited growth of *Alternaria brassicae* in co-cultured experiments, whereas targeted gene deletion of *BbAFP1* significantly decreased (25%) this inhibitory effect. Recombinant BbAFP1 showed chitin and glucan binding abilities, and growth inhibition of a wide range of phytopathogenic fungi by disrupting membrane integrity and eliciting reactive oxygen species (ROS) production. A phenylalanine residue (F^50^) contributes to chitin binding and antifungal activity, but was not required for the latter. Expression of BbAFP1 in tomato resulted in transgenic plants with enhanced resistance to plant fungal pathogens. These results highlight the importance of fungal competition in shaping primitive competition strategies, with antimicrobial compounds that can be embedded in the spore cell wall to be released into the environment during the critical initial phases of germination for successful growth in its environmental niche. Furthermore, these peptides can be exploited to increase plant resistance to fungal pathogens.

## Author summary

Microbial competition exerts powerful selective pressures for the development of defensive and offensive methods of suppressing potential competitors. One of the most vulnerable stages for any fungi is the initial germination of resting spores in potentially hostile environments. Currently, we know little about how fungi defend other microbial competitors during the beginning stage of conidial germination. Here, we report on an antifungal peptide from *B*. *bassiana* (BbAFP1) that is specifically expressed in mature aerial conidia, with the protein localized exclusively to the conidial cell wall. The “pre-loaded” BbAFP1 is released into the surrounding microenvironment where it can act to inhibit the growth of competing fungi during the initial stages of fungal germination, i.e. largely before actual germ tubes are apparent, thus conferring an advantage to *B*. *bassiana* in out-competing susceptible competitors in the microenvironment surrounding the spore. The effects of BbAFP1 on membrane integrity were characterized and a key amino acid (F^50^) was shown to function in chitin binding and antifungal activity. Transgenic tomato overexpressing BbAFP1 were shown to exhibit enhanced resistance to plant fungal pathogens. Our study provides new insights into the microbial competition and genes involved in this process that can be exploited to increase plant disease resistance.

## Introduction

The production of antimicrobial peptides (AMPs) represents a ubiquitous initial defense and/or offensive mechanism for inhibiting the growth of competitors. Largely characterized in animals, plants, and bacteria, only ~1% of known AMPs are from fungi [1]. In the former two (i.e. higher eukaryotes), AMPs function in resistance to microbial infections as part of innate immune systems, and most are amphipathic cationic molecules whose net positive charge promotes binding to (negatively charged) microbial membranes, where they typically, though not always, compromise the integrity (structural and/or functional) of target membranes resulting in growth inhibition and/or cell death [2, 3]. Within the context of higher eukaryotes, many AMPs are induced in response to invading microbes via sometimes elaborate sensing and signal transduction pathways [4, 5]. Spanning a wide variety, but often related, three-dimensional structures, functional targets, and richness and distribution of certain amino acids (e.g. glycines, cysteines, proline, arginines, and lysine), AMPs are classified using a range of such properties, and cross-reactivity of AMPs displaying both antibacterial and antifungal effects have been noted [6–9]. Although few AMPs (and even fewer antifungal proteins, AFPs) have been characterized in fungi, comparative genomic analyses have revealed the phylogenetically diversified distribution of closely related AMPs, supporting the idea of the ancient ancestral occurrence of these peptides in basal eukaryotic lineages [10]. A conserved γ-core motif with antifungal activity has been identified in AFPs from ascomycetes, which shows the potential as a motif for the design of short antifungal peptides [11]. In addition, the identification of a fungal defensin, consisting of a stabilized α-helix and β-sheet structural motif (CSαβ), named plectasin, with high activity towards *Streptococcus pneumoniae*, has led to the categorization of six broad families of fungal defensin-like peptides [10, 12]. However, again, the specificities (antibacterial versus antifungal) and/or biological functions of only a handful of these are known.

A number of small, basic antifungal proteins secreted by filamentous fungi have been characterized [13]. These include lfAFP from *Aspergillus giganteus*, Anafp from *A*. *niger*, NFAP and NFAP2 from *Neosartorya fischeri* and PAF and PAFB from *Penicillium chrysogenum*. AfpA and AfpB from *P*. *digitatum*, and PeAfpA, AfpB, and AfpC from *P*. *expansum* [14–21], all of which have been shown to exhibit some inhibitory activity towards a range of (other) filamentous fungi. In addition, structural information concerning several of these, e.g. PAF, PAFB, NFAP and NFAP2, are available [17, 22–27]. These proteins contain signal peptides sites at the N-terminus, although the exact nature of the processing remains obscure. Tertiary structural studies have revealed that AFP consists of a β barrel of five antiparallel β strands stabilized by cysteine mediated disulfide bridges, that confer to the protein high (temperature and pH) stability [22, 28]. PAF appears to be able to inhibit fungal (*A*. *nidulans*) growth by disrupting plasma membrane integrity and inducing apoptosis [29]. PAF has been described as morphogenic, in that it induces severe changes in the morphology of its targets. In contrast, AFP appears to be non-morphogenic, and one suggested the mechanism of its action is via direct interaction with the target plasma membrane and pore formation, potentially via interactions with specific receptors on sensitive hosts [13]. It was also been found that AFP can bind to chitin and inhibit chitin synthase activity in the cell wall of sensitive filamentous fungi [30]. However, to date, the cellular localization and the nature of the biological/environmental mechanisms of the functioning of these AFPs have yet to be reported.

*Beauveria bassiana* is known as a broad host range entomopathogenic fungus, widely used as a microbial insecticide for the biological control of agricultural, forest, and urban pests [31]. However, in addition to parasitizing insect hosts, *B*. *bassiana* has other lifestyles, and can grow as a saprophyte, exists in the plant phylloplane, and appears to form endophyte-like relationships with several plants [32–34]. Within this ecological context, *B*. *bass*iana has been shown to potentially act as an antagonist to plant pathogenic fungi and/or promote plant growth [35]. Competition bioassays have shown that *B*. *bassiana* can inhibit the growth of a number of plant fungal pathogens, including *Gaeumannomyces graminis*, *Fusarium oxysporum*, *Botrytis cinerea*, and *Rhizoctonia solani* [36]. In addition, seed application of *B*. *bassiana* protected tomato and cotton seedlings against *R*. *solani* and *Pythium myriotylum* [37]. However, the underlying genetic mechanism(s) responsible for these activities have yet to be reported. The diverse ecological niches occupied by *B*. *bassiana* necessitates contact with a diverse range of competing microbes [38], however, again, little is known concerning the genetic mechanisms that have evolved to successfully compete in these environments. The characterization of the production of the secondary metabolite oosporein, as an antibacterial agent specifically after the death of the host, in order to maximally utilize the nutrient on the cadaver and successfully sporulate is one exception [39]. Here, we have identified a gene encoding an antifungal peptide in *B*. *bassiana BbAFP1*. *BbAFP1* was specifically expressed in mature aerial conidia and the protein was localized to the conidial cell wall. We further show that as conidia germinate the peptide is released into the surrounding microenvironment, where it can inhibit the growth of competing fungi. Site directed mutagenesis of key amino acid residues of BbAFP1 identified a phenylalanine (F^50^) important for mediating both chitin binding and antifungal activity. *B*. *bassiana* targeted gene knockout and over-expression strains showed reduced and enhanced abilities to compete with various fungi, respectively. Transgenic expression of BbAFP1 in tomatoes resulted in increased antifungal resistance in the plants. These data demonstrate the existence of a competition factor that acts to help fungal conidiospores confront competing fungi during the initial stages of spore germination and growth.

## Results

### BbAFP1 inhibits the germination and growth of filamentous fungi

**Fig 1:**
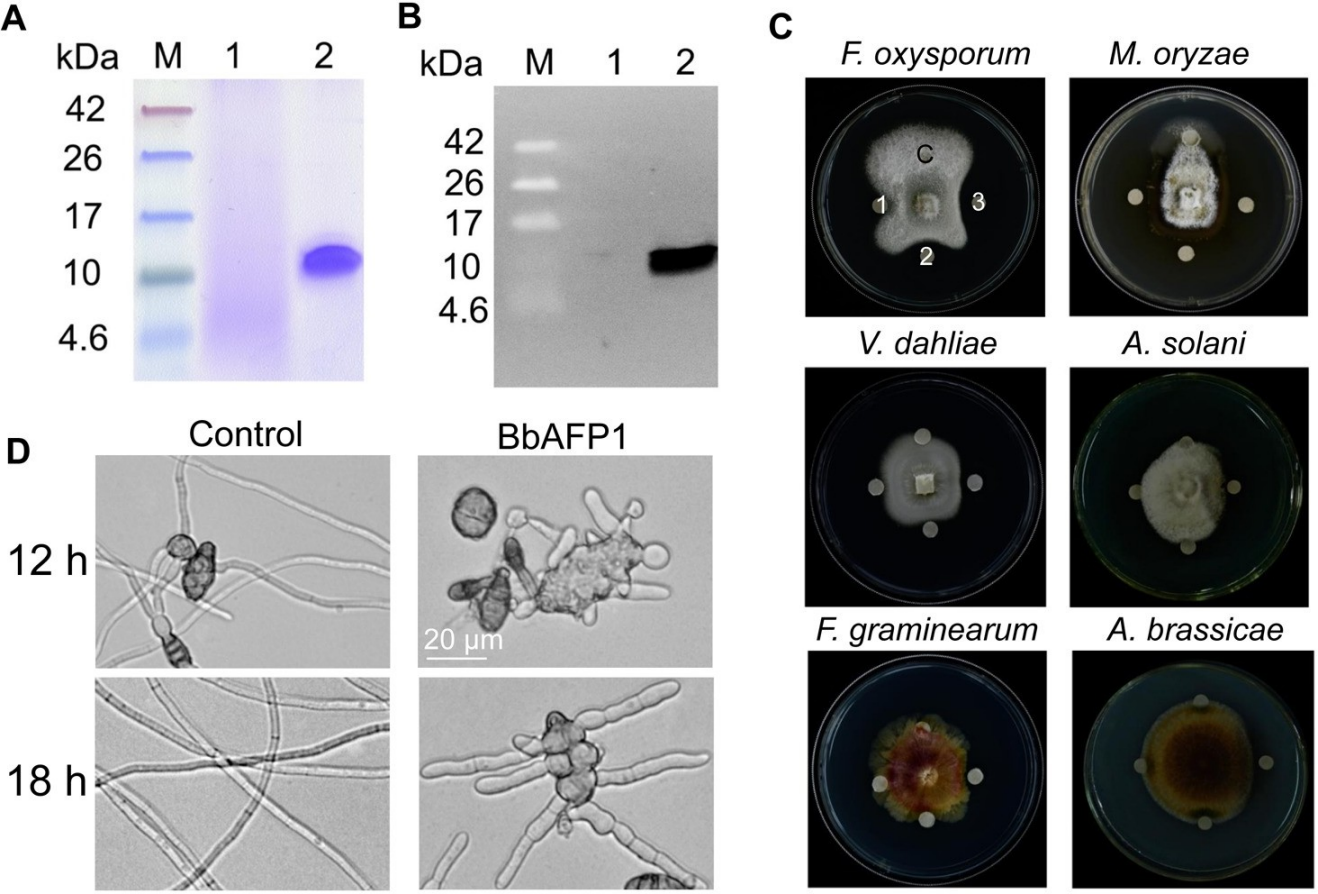
Purification and inhibitory activity of BbAFP1 against filamentous fungi. (A) SDS-PAGE and (B) Western blot analyses of BbAFP1 expressed in *P*. *pastoris*. M, prestained low molecular weight markers. Lane 1, supernatant of *P*. *pastoris* transformed with pPIC9k (control); Lane 2, BbAFP1 purified from *P*. *pastoris* by His-tag affinity chromatography. (C) Effects of BbAFP1 on growth of indicated fungi on PDA plates. Labels on plate are as follows; C = control, 20 mM NaAC (pH 5.4). 1–3 = 0.5, 1.0, 1.5 μg of BbAFP1 spotted onto disks, respectively. The same sequence of samples was used in all plates. (D) Representative images of the effects of BbAFP1 on conidial germination and hyphal growth of *A*. *brassicae*. A conidial suspension of *A*. *brassicae* (1 × 10^6^ conidia/ml) was inoculated into PDB containing 5.0 μM BbAFP1 and cultured at 26°C for 12–18 h. The amount of BbAFP1 was determined by the Bradford method.

A survey of the *B*. *bassiana* genome identified an open reading frame (ORF), designated as *BbAFP1* that could be translated into an 89 amino acid peptide that showed high (73–93%) sequence identity to putative antifungal proteins found in various filamentous fungi (S1 Fig). Phylogenetic analyses indicated that the antifungal proteins separated into at least 4 clades, with the *B*. *bassiana* protein belongs to PAF-cluster. A 19-a.a. signal peptide was identified at the N-terminus, suggesting the protein is processed and secreted, with the mature protein contained 6 cysteine with 8 arginine and 3 lysine residues, pI = 9.01 (S1 Fig and S1 Table). To confirm the antifungal activity of BbAFP1, the protein without native signal peptide sequence but with yeast α-factor secretion signal peptide and a C-terminal 6 × histidine tag, was expressed in a yeast, *Pichia pastoris*, heterologous expression system. The protein was purified from *P*. *pastoris* using nickel affinity chromatography as detailed in the Methods section (Fig 1A and 1B). Antifungal activity of purified BbAFP1 was analyzed by disk diffusion assays examining inhibition of growth to various fungi. BbAFP1 displayed highest antifungal activity towards *Fusarium oxysporum*, followed by *F*. *gramminearum*, *Magnaporthe orzyae*, *A*. *brassicae*, *Verticillium dahliae*, *A*. *solani*, and with minimum inhibitory concentrations (MIC) = 5–15 μM (Fig 1C and Table 1). However, no significant growth inhibition was observed when BbAFP1 was tested against bacteria and yeast (S2 Fig). To further evaluate the effects of the BbAFP1 on fungal germination and growth, the purified protein (5 μM) was added to *A*. *brassicae* conidial suspensions. Conidial germination (> 90%) was seen 12 h post-inoculation in control samples, with extensive hyphal growth visible within 18 h in PDB liquid media. In contrast, in the presence of BbAFP1, only a few (< 30%) *A*. *brassicae* conidia germinated within 12 h, with those that did forming swollen, distorted, abnormal-branched germ tubes that failed to elongate further over time (up to 18 h) (Figs 1D and S3).

**Table 1:** MICs of BbAFP1 and BbAFP1^F50A^ for selected microorganisms.

Organism	MIC (μM)	
	BbAFP1	BbAFP1^F50A^	
*A*. *brassicae*	10	35–40	
*A*. *solani*	10–15	40	
*F*. *oxysporum*	5	20–25	
*F*. *graminearum*	7.5–10	30–35	
*Trichoderma* sp.	5–7.5	25	
*V*. *dahliae*	10–15	35–40	
*B*. *cinerea*	10–15	35–40	
*M*. *oryzae*	7.5–10	30	
*Penicillium* sp.	> 40	> 40	
*B*. *Bassiana* 0062	> 40	> 40	
*B*. *bassiana* ATCC 90517	> 40	> 40	

**Fig 2:**
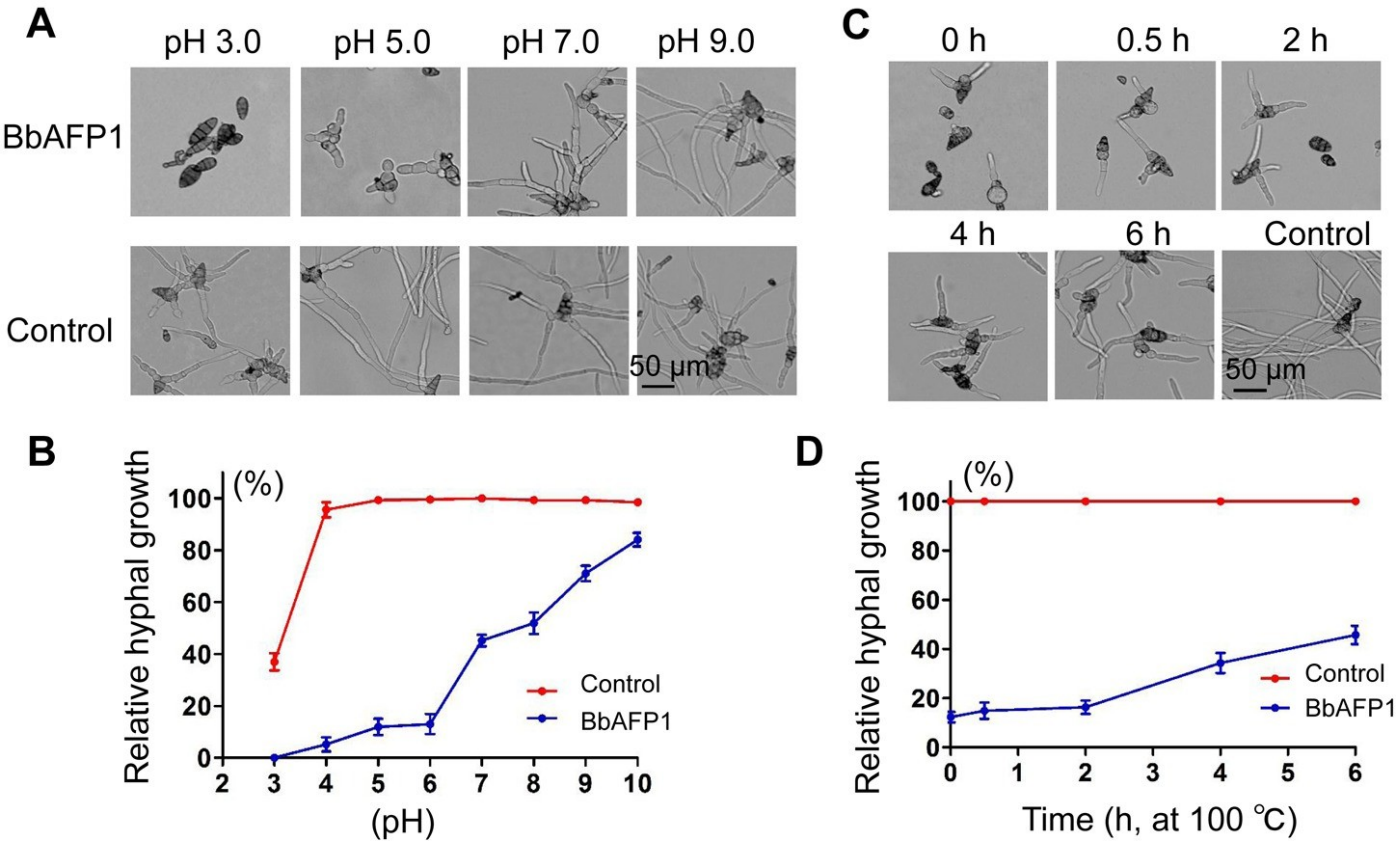
Properties of BbAFP1. (A, B) Effects of pH and (C, D) high temperature on BbAFP1 antifungal activity using *A*. *brassiacae* as the target. Growth of *A*. *brassiacae* at 26℃ in 20 mM NaAC (pH 5.4) without BbAFP1 was used as the standard condition and set = 100% Relative hyphal growth under test conditions was calculated as [hyphal length^treatment^]/[hyphal length^standard condition^] × 100%. Error bars = SD.

The antifungal activity of BbAFP1 as a function of pH was examined. When normalized to the standard growth condition, 26°C, 20 mM NaAC, pH 5.4, with *A*. *brassicae* as the test fungus, BbAFP1 showed higher growth inhibitory activity under acidic conditions, with a relative hyphal growth of approximately 15%. However, antifungal activity dramatically decreased under alkaline conditions (pH 8.0–10.0, relative hyphal growth > 50%) (Fig 2A and 2B), indicating that the antifungal activity of BbAFP1 is optimal under acidic conditions. Thermostability analysis showed that the antifungal activity of BbAFP1 was not affected by treatment at 100°C for up to 2 h, with only slight decreases (20–30%) after treatment for 4–6 h (Fig 2C and 2D).

### BbAFP1 depolarizes target membranes and results in the production of a reactive oxygen species (ROS) burst

**Fig 3:**
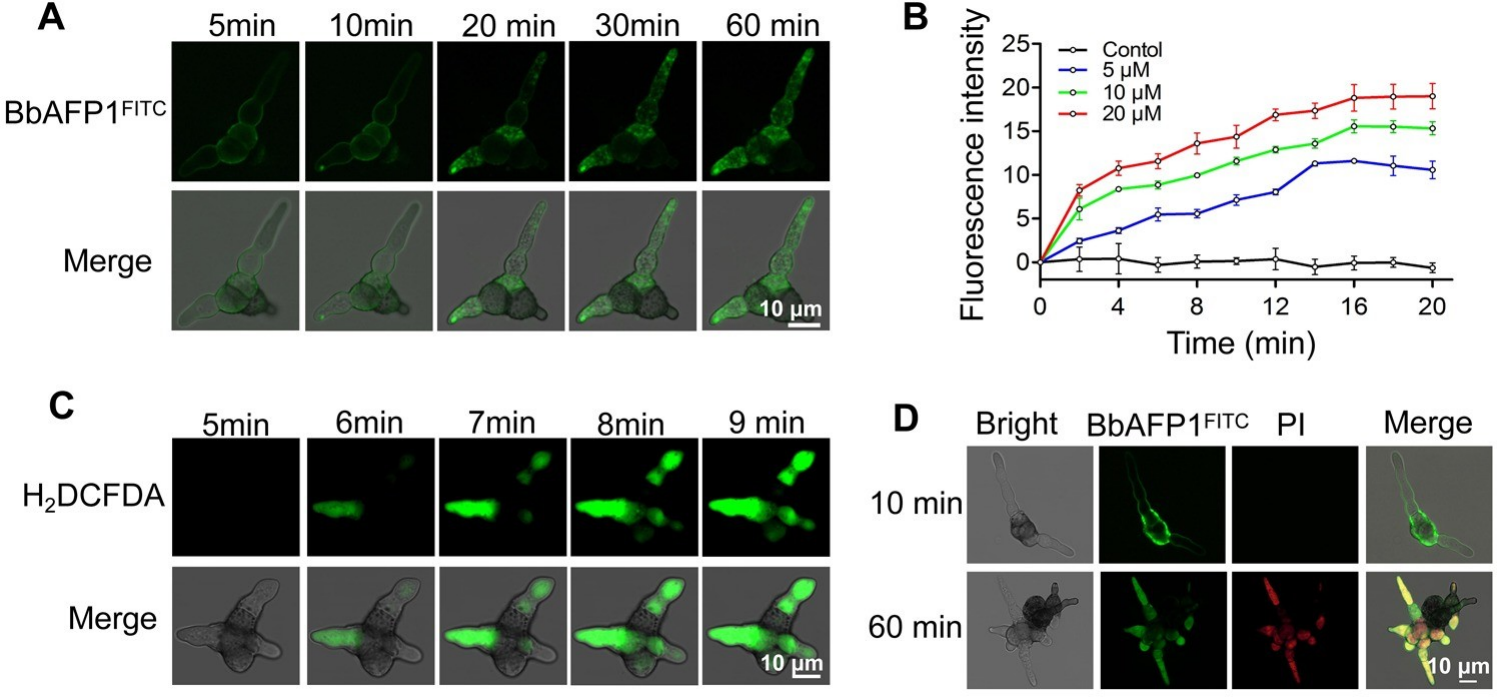
BbAFP1 depolarizes target membranes and results in the production of a reactive oxygen species (ROS) burst. (A) Dynamic processes of BbAFP1 entering target fungal cells. Pre-germinated *A*. *brassicae* conidia were treated with BbAFP1^FITC^ and the protein internalization was observed (5–60 min). (B) Effects of BbAFP1 on membrane potential change in *A*. *brassicae* cells using DiSC3(5) staining. After treated germinated *A*. *brassicae* conidia with membrane potential-sensitive dye DiSC3(5), BbAFP1 (5–20 μM) was added to the cells and the fluorescence intensity was detected (2–20 min). (C) Effects of BbAFP1 on ROS burst using H_2_DCFDA staining. (D) Propidium iodide (PI) uptake analysis. After treated *A*. *brassicae* cells with BbAFP1^FITC^ and PI solution for 10 min or 60 min, fluorescent signals were observed. In these experiments, *A*. *brassicae* cells were pre-cultured at 26°C for 5 h in PDB. All experiments were performed in triplicate with at least three independent biological samples. Error bars = SD.

In order to probe the potential mechanisms of BbAFP1 activity, the protein was labeled with FITC as detailed in the Methods section. After addition of BbAFP1^FITC^ to *A*. *brassicae* cells, a strong fluorescent signal was enriched on the surfaces of cells within the first 10 minutes. Subsequently, the fluorescent signal appeared inside the cells and enhanced gradually (Fig 3A and S1 Video). The effect of BbAFP1 on fungal cell membrane potential was analyzed using the voltage-sensitive dye 3,3′-dipropylthiadicarbocyanine iodide DiSC3(5). Treatment BbAFP1 resulted in the rapid depolarized of the *A*. *brassicae* cell membrane potential in a concentration dependent manner. A rapid increase in DiSC3(5) release was seen within the first few minutes, with a gradual plateau reached within 16 min (Fig 3B). Detection of the production of reactive oxygen species (ROS, with the substrate H_2_DCFDA), resulted in rapid bursts of ROS production within 5–9 min after addition of BbAFP1 to cells (Fig 3C and S2 and S3 Videos). Membrane disruption and ultimately cell death was further visualized by staining of BbAFP1^FITC^ treated *A*. *brassicae* cells with propidium iodide (PI). These results showed an initial BbAFP1^FITC^ membrane staining (10 min), after which within 60 min post-treatment, the BbAFP1^FITC^ and PI signals were co-localized inside the fungal cells (Fig 3D). After 3 h post-treatment, protein and nucleic acids could be detected in the supernatant of BbAFP1-treated cells (S4 Fig).

**Fig 4:**
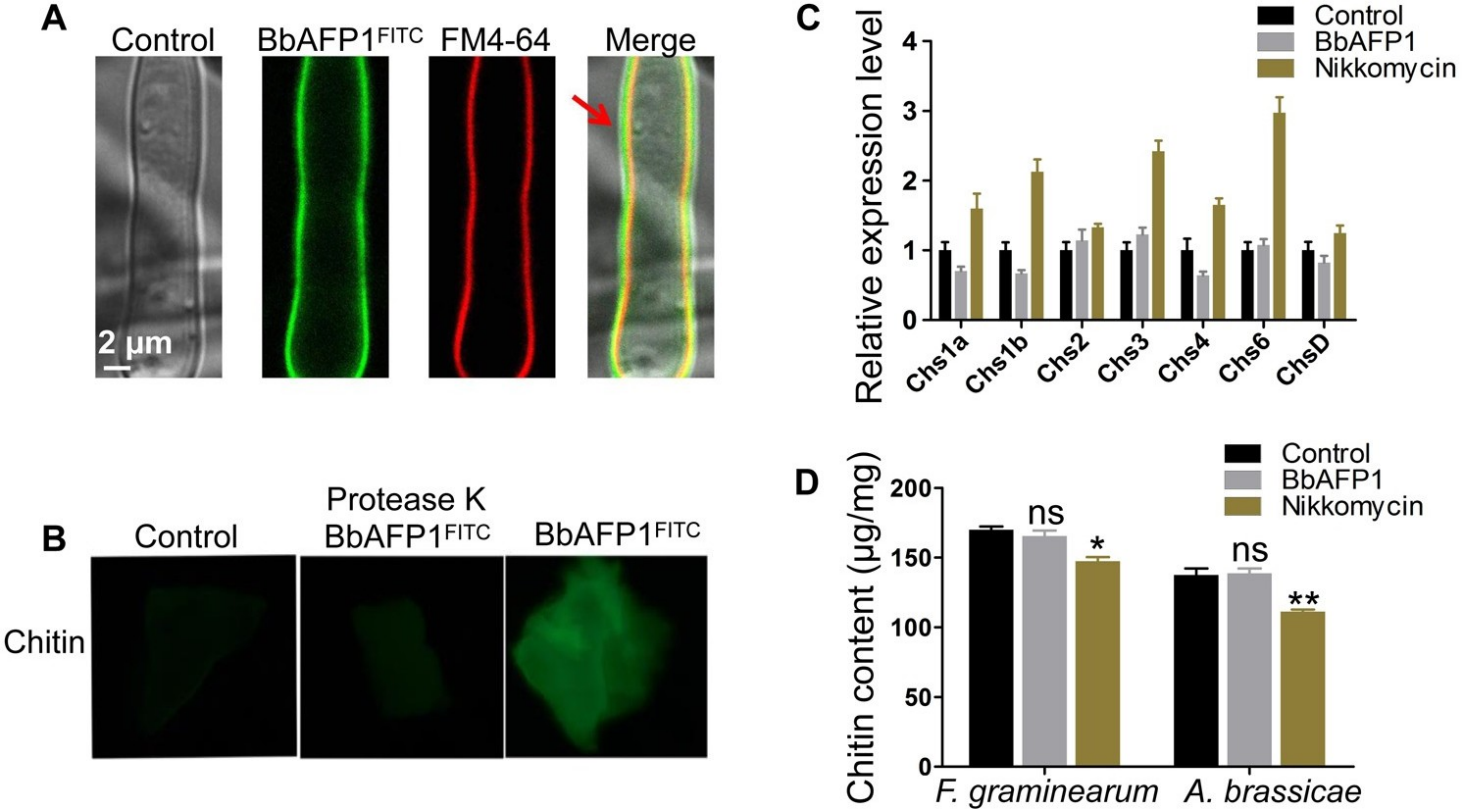
Chitin binding ability of BbAFP1 and its effects on chitin synthesis. (A) Binding of BbAFP1^FITC^ to cell wall of *A*. *brassicae*. *A*. *brassicae* cells pre-cultured at 26°C for 10 h in PDB were treated with BbAFP1^FITC^ and membrane dye FM4-64 for 10 min and fluorescent signals were observed by confocal microscopy. (B) Binding of BbAFP1^FITC^ to chitin. Powdered chitin treated with 20 mM potassium phosphate buffer (pH 6.0) was used as a control. After binding of BbAFP1^FITC^, protease K was added to digest the proteins bound in chitin. (C) Effects of BbAFP1 on the expression level of chitin synthase genes of *F*. *graminearum*. After treated *F*. *graminearum* with BbAFP1 or chitin synthesis inhibitor (nikkomycin) for 2 d, total RNA was isolated and RT-PCR analysis was performed with β-*tubulin* as the reference gene. (D) Effects of BbAFP1 on chitin contents in fungal cell wall. After treated with BbAFP1 or nikkomycin for 2 d, mycelium of *A*. *brassicae* and *F*. *graminearum* were collected and lyophilized. Chitin contents of mycelium were measured based on N-acetylglucosamine standard curve. NaAC (20 mM, pH 5.4) was used as a control. All experiments were performed in triplicate with at least three independent biological samples. Error bars = SD, ns indicates no significance, * indicates 0.01 < P < 0.05, ** indicates P < 0.01, t-test.

In order to verify the initial fungal membrane binding of BbAFP1, co-localization analyses using the membrane staining dye FM4-64, revealed overlapping signals at the cell periphery (Fig 4A). *In vitro* binding assays revealed BbAFP1 was able to bind to chitin (Fig 4B), however, no effects on the expression of chitin synthesis genes and/or overall chitin content in the cell walls of treated fungi (either *A*. *brassicae* or *F*. *graminearum*) was noted, whereas treatment with nikkomycin, known to inhibit overall chitin cell wall content [40] induced expression of several chitin synthases but resulted in an overall net decrease in chitin content (Fig 4C and 4D). Intriguingly, BbAFP1^FITC^ was able to bind to *B*. *bassiana* conidia, but not to germinated conidia (S5 Fig).

### Chitin binding and antifungal activity are mediated in part by a critical phenylalanine residue (F^50^)

**Fig 5:**
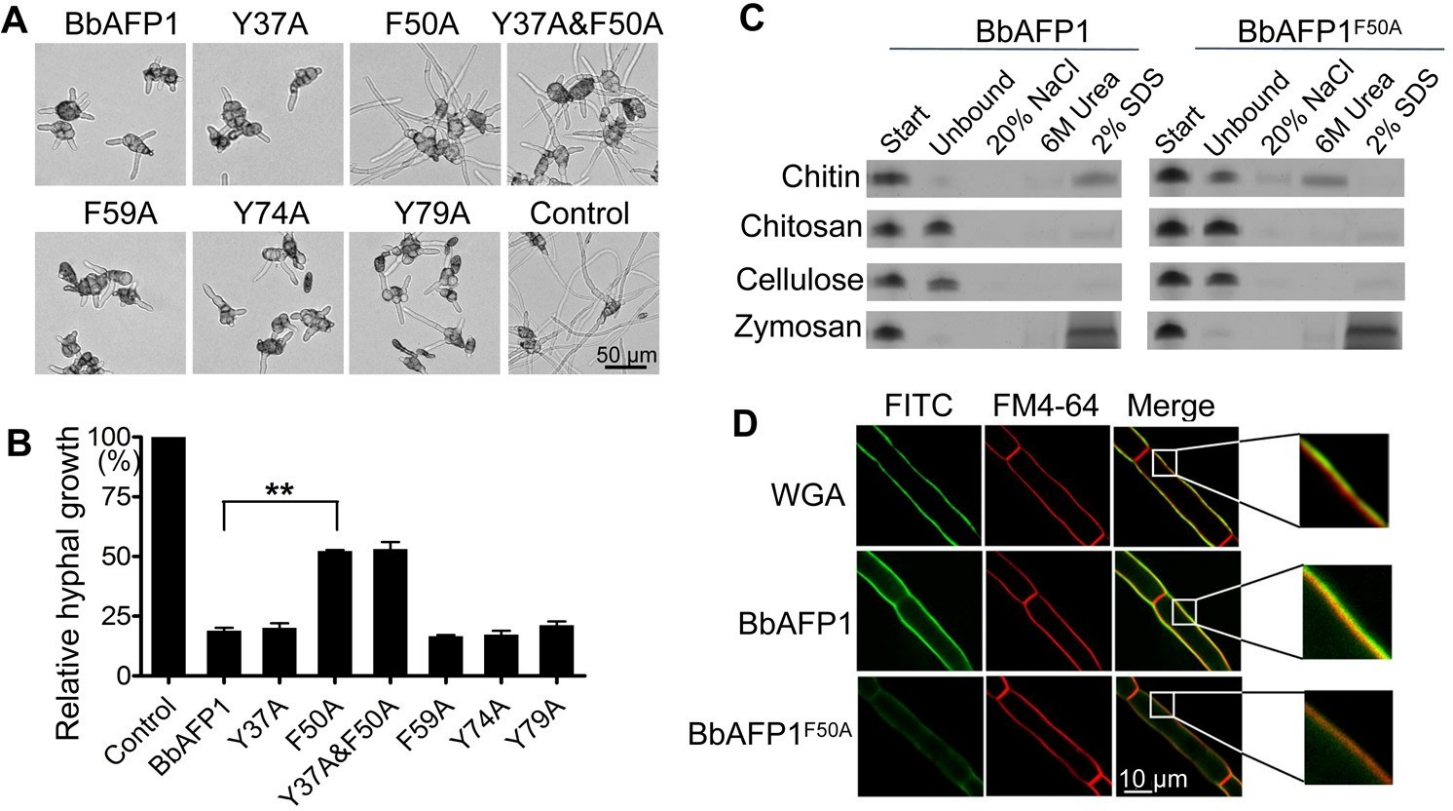
Mutational analysis of BbAFP1. (A, B) Representative images and relative antifungal activity of BbAFP1 mutants. BbAFP1^F50A^ and BbAFP1^Y37A&F50A^ mutants showed decreased effectiveness in inhibiting growth of *A*. *brassiacae*. All other tested mutants were similar to the wild type protein in ability to inhibit *A*. *brassiacae* germination and growth. (C) SDS-PAGE analyses of binding and elution of BbAFP1 and BbAFP1^F50A^ to different substrata including chitin, chitosan, cellulose, and zymosan. (D) Cellular localization analysis of BbAFP1^FITC^ and BbAFP1^F50A^-^FITC^ on hyphae of *A*. *brassicae*. FITC labelled protein was quantified by Bradford method and 2.5 μM protein was added into the hyphae germinated from 600 μl *A*. *brassicae* cells (1 × 10^6^ conidia/ml). WGA and FM4-64 were used as cell wall and cell membrane counter stains, respectively. All experiments were performed in triplicate with at least three independent biological samples. Error bars = SD, ** indicates P < 0.01, t-test.

An amino acid alignment of BbAFP1 with other characterized fungal AMPs confirmed the locations of conserved cysteine residues as well as many of the positively charged (arginine and lysine) residues (S1 Fig). Intriguingly, however, BbAFP1 appeared to be particularly enriched in aromatic amino acids with three tyrosine and two phenylalanine residues. In order to probe the potential functional consequences of this enrichment, a series of site directed mutant of BbAFP1 including Y^37A^, F^50A^, F^59A^, Y^74A^, Y^79A^, and Y^37A^ F^50A^ double mutant were generated, expressed and purified from *P*. *pastoris*. No significant decrease in antifungal activity as compared to wild type BbAFP1 was observed for mutants Y^37A^, F^59A^, Y^74A^, and Y^79A^. In contrast, the antifungal activities of the single mutant BbAFP1^F50A^ and the double mutant BbAFP1^Y37A&F50A^ were markedly decreased, with antifungal activity decreasing ~50% as compared to the wild type BbAFP1 or any of the other mutants (P < 0.01, Fig 5A and 5B). The MIC of mutant BbAFP1^F50A^ against sensitive fungi was increased ~3-5-fold compared to wild-type protein (Table 1). The BbAFP1^F50A^ mutant also showed altered binding to powdered chitin. Whereas the wild type protein could be eluted from the chitin only by boiling in 2% SDS, a smaller fraction of the BbAFP1^F50A^ bound to the chitin could be eluted by 6 M urea and to a lesser extent by 20% NaCl (Fig 5C). Compared to BbAFP1^FITC^, fluorescence signal on powdered chitin treated with BbAFP1^F50A^-^FITC^ was obviously reduced (S6 Fig). Co-localization analysis showed that BbAFP1^FITC^ localizes to the cell wall and cell membrane of *A*. *brassicae*, whereas the BbAFP1^F50A^-^FITC^ showed reduced cell wall and membrane binding (Fig 5D). However, no differences in term of cell wall binding ability and/or inhibitory action against *Phytophora nicotianae* (whose cell wall does not appear to contain chitin), were observed between BbAFP1 and BbAFP1^F50A^ (S7 Fig). Neither protein (wild type or the BbAFP1^F50A^ mutant) showed appreciable binding to chitosan or cellulose, whereas both proteins bound to zymosan and could only be eluted using 2% boiling SDS. Direct glucan binding was also observed using BbAFP1^FITC^ against glucan particles (S8A Fig). RT-PCR analysis showed that the expression of two glucan synthesis related genes, 1,3-beta-glucan synthase and the glucan synthesis regulatory protein gene, are not affected after treatment of *F*. *graminearum* with BbAFP1. However, they both genes were significantly (P < 0.01) upregulated when treated with the chitin synthesis inhibitor, nikkomycin (S8B Fig).

### BbAFP1 is specifically expressed in *B*. *bassiana* conidia and is localized to the cell wall and can be released into the surrounding microenvironment

**Fig 6:**
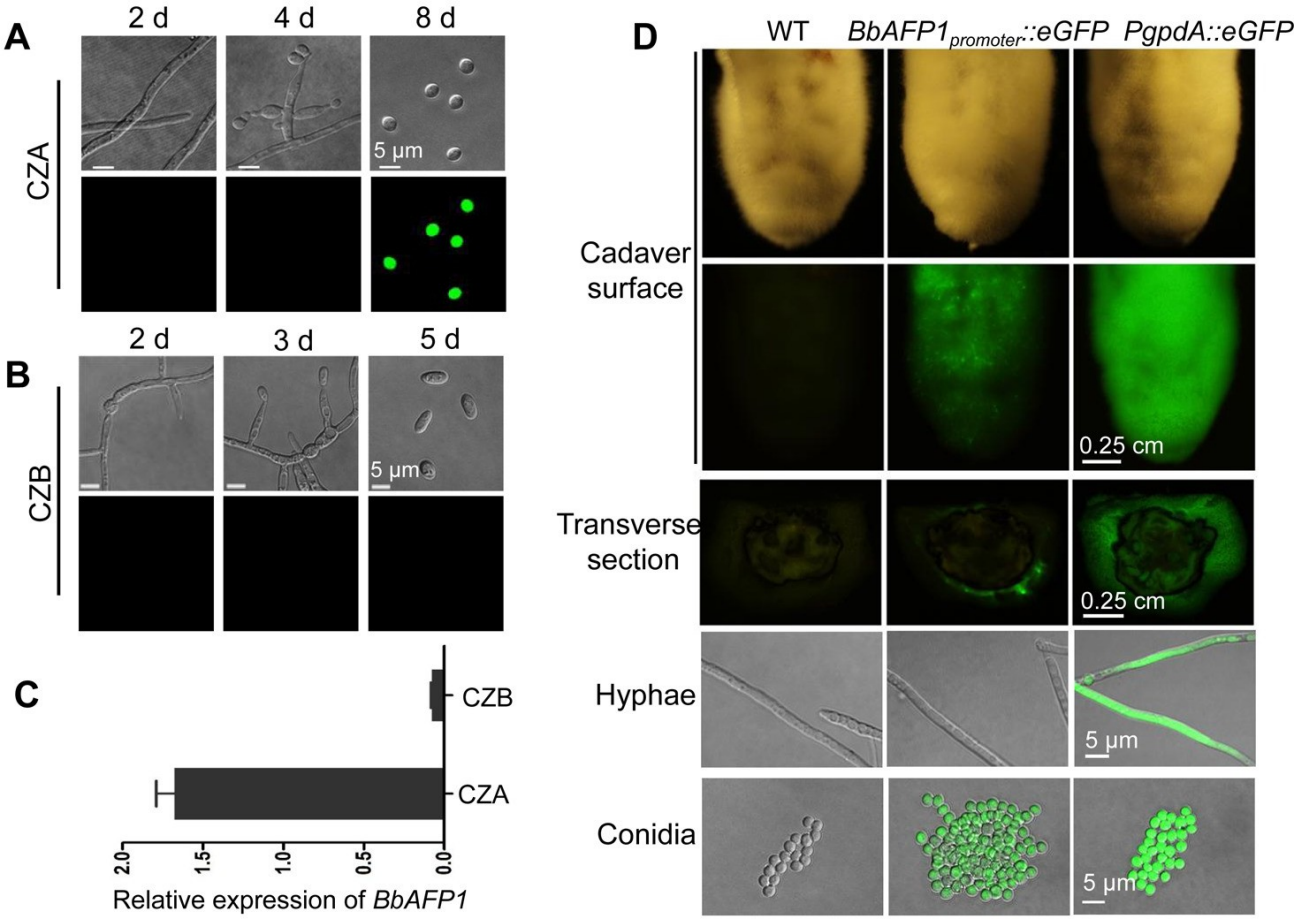
Representative images of *BbAFP1*_*promoter*_::*eGFP* expression in *B*. *bassiana*. (A, B) Expression of *BbAFP1*_*promoter*_::*eGFP* in *B*. *bassiana* cultured on CZA (Czaped-Dox agar) (2, 4, and 8 d) or in CZB (Czaped-Dox broth) (2, 3, and 5 d), respectively. (C) RT-PCR quantification of *BbAFP1* expression in conidia from CZA (8 d) and blastospore from CZB (4 d). (D) Representative images of *BbAFP1*_*promoter*_::*eGFP* expression in hyphae and conidia produced on *G*. *mellonella* cadavers. Wild type (no signal) and a strain constitutively expressing eGFP (using the *B*. *bassiana PgpdA* promoter, no fusion protein), were used as negative and positive controls, respectively. Error bars = SD.

In order to probe the conditions under which BbAFP1 is expressed, a green fluorescent protein (eGFP)-BbAFP1-promoter (gene expression) construct, was transformed into wild type *B*. *bassiana* (to yield the *BbAFP1*_*promoter*_::*eGFP* strain). As BbAFP1 exhibits antifungal ability, we first hypothesized that this gene may require the presence of competing (antagonistic) fungi for expression. However, no GFP signal was seen in growing *B*. *bassiana* hyphae either in the presence or absence of various competing fungi including *A*. *brassicae*, *B*. *cinerea*, or *V*. *dahliae* in/on liquid or solid medium (S9 Fig). Analysis of GFP fluorescence in the *BbAFP1*_*promoter*_::*eGFP* strain in CZA revealed no significant signal in growing hyphae (2 d post-inoculation of conidia) or during the initial/early stages of conidiation (4 d growth), but a strong signal could be seen in mature aerial conidia (> 8 d) (Fig 6A). No (GFP) signal was detected in CZB (liquid) media in hyphae, submerged conidia and/or blastospores (Fig 6B). These results were confirmed via real-time quantitative PCR (Fig 6C). To further analyze the expression of *BbAFP1* during infection, larvae of the greater wax moth, *Galleria mellonella*, were topically (the “natural” means of infection) treated with *BbAFP1*_*promoter*_::*eGFP* conidia. No GFP signal was detected in the *in vivo* hyphal bodies isolated from infected insect hemolymph or in the hyphae formed both inside and on the host cadaver post host death (S10 Fig). However, a robust GFP signal was observed in conidia produced on infected *G*. *mellonella* cadavers during the final stages of conidial production (Fig 6D).

**Fig 7:**
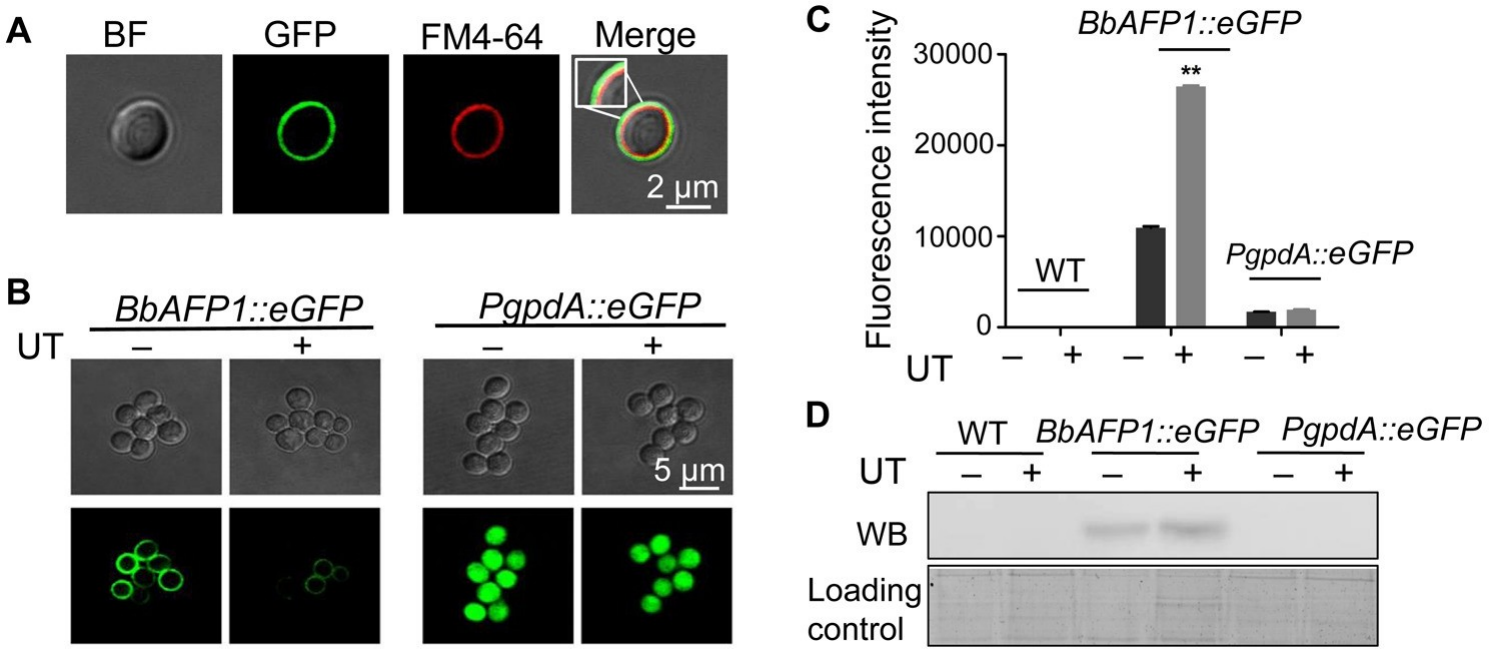
Conidial cell wall localization of BbAFP1. (A) Representative images of conidia of the *BbAFP1*_*promoter*_::*BbAFP1*::*eGFP* strain counter stained with the membrane dye FM4-64. (B) Representative images of release of BbAFP1::eGFP from conidia by ultrasonic treatment (UT). Control experiments were performed using the constitutive expressing *PgpdA*::*eGFP* strain. (C) Detection of solubilized BbAFP1::eGFP via fluorometry in the supernatant of conidial suspensions (*BbAFP1*_*promoter*_::*BbAFP1*::*eGFP*) treated by ultrasonic treatment. Control experiments were performed using the wild type and constitutive expressing *PgpdA*::*eGFP* strains. (D) Western blot analyse of BbAFP1::eGFP in the conidial supernatant after ultrasonic treatment. Proteins were transferred to membrane and probes with an anti-GFP antibody. UT indicates ultrasonic treatment. All experiments were performed in triplicate with at least three independent biological samples. Error bars = SD, ** indicates P < 0.01, t-test.

As our data indicated that BbAFP1 is expressed in aerial conidia and possesses a secretory signal peptide (as predicted by Signal P), we were interested in determining the subcellular localization of the protein in *B*. *bassiana* conidia. In order to examine this, a fusion protein consisting of eGFP linked to the C-terminus of BbAFP1 under the control of its native promoter was constructed and transformed into *B*. *bassiana* to yield the *BbAFP1*_*promoter*_::*BbAFP1*::*eGFP* strain. As seen with the *BbAFP1*_*promoter*_::*BbAFP1*::*eGFP* strain, the GFP signal was observed exclusively in mature aerial conidia and could be seen distinctly staining the cell envelope of conidia and only appeared in the mature conidia as observed in promoter reporter strain *BbAFP1*_*promoter*_::*eGFP* (S11 Fig). To better validate the cell-wall localization, counterstaining with the membrane dye FM4-64, showed BbAFP1 mainly localizes to the outer cell wall layer (i.e. outside of the cell membrane, Fig 7A). When *BbAFP1*_*promoter*_::*BbAFP1*::*eGFP* bearing conidia were treated to mild sonication, the GFP signal was lost in conidia and was recovered in the cell-free supernatant as determined by fluorescence intensity detection and Western blot analysis (Fig 7B–7D). However, under the same conditions, the GFP signal was not affected in conidia from the control *PgpdA*::*eGFP*, and no GFP signal was released into solution.

**Fig 8:**
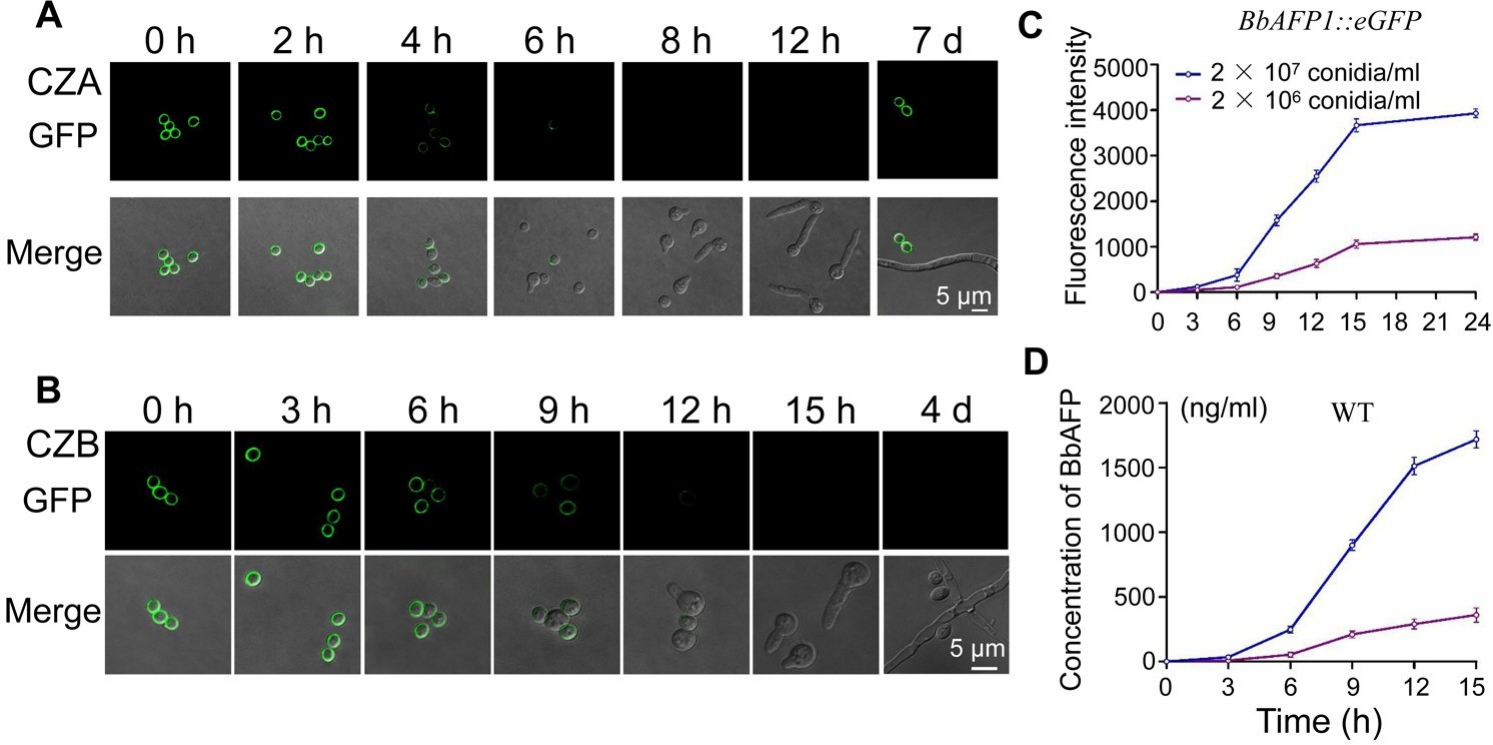
Representative images of developmental time course of BbAFP1::eGFP expression and localization in *B*. *bassiana*. (A, B) Fluorescent images of *B*. *bassiana* cells expressing BbAFP1::eGFP after inoculation on CZA or in CZB, respectively. (C) Fluorometric quantification of BbAFP1::eGFP in the culture media (cell-free supernatant) over the indicated time course after inoculation in CZB. (D) Quantification of BbAFP1 production in the culture media (cell-free supernatant) of wild type *B*. *bassiana* over the indicated time course after inoculation in CZB as determined by ELISA. All experiments were performed in triplicate with at least three independent biological samples. Error bars = SD.

The expression/localization was also examined in germinating aerial conidia (no sonication or other treatment). Freshly harvested conidia displayed a strong cell wall signal that was retained 2 h post-inoculation onto (agar) nutrient media (CZA), that was noticeably reduced 4 h post-inoculation (but before any germination was noticeable) (Fig 8A). By 6 h post-inoculation, with < 10% germination visible, the signal was almost completely lost, and no further signal could be detected during germination and germ tube/hyphal growth until newly produced aerial conidia were visible (Fig 8A). Similar results were seen when conidia were inoculated into liquid nutrient media, BbAFP1::eGFP remained associated with the conidia up to 6 h post-inoculation in CZB, after which the signal was gradually lost from the cells (Fig 8B). By the time the spores were germinating (12 h), very weak signal could be detected on the cells, and when germ tubes were clearly visible on most cells (> 80% germination, 15 h post-inoculation), almost no fluorescence was seen on the cells. In addition, the blastospores or submerged conidia produced under these conditions at 4 d post-growth failed to show expression/localization of BbAFP1::eGFP to the membranes of these cells (Fig 8B and 4 d panel). The loss of signal on the conidial surface correlated with a concomitant recovery of the signal in the culture broth as measured either directly (fluorescence intensity in culture supernatant, Fig 8C) or by ELISA using the BbAFP1 antibody (Fig 8D), with the signal recovered proportional to the initial conidial concentration used.

### Construction and characterization of *BbAFP1* targeted gene knockout (Δ*BbAFP1*) and over-expression (*BbAFP1*^*OE*^) strains

To further probe the biological functions of BbAFP1, targeted gene knockout (**Δ***BbAFP1*) and overexpression (*BbAFP1*^*OE*^) strains were constructed as detailed in the Methods section (S12 Fig). *BbAFP1* overexpression was achieved via use of the constitutive *B*. *bassiana* glyceraldehyde 6-phosphate dehydrogenase (*gpdA*) promoter driving expression of *BbAFP1*. Relative expression levels of *BbAFP1* was examined in various transformants, and the clone displaying the highest level was chosen for further study (S12 Fig). At 16 h post inoculation, in situ, i.e. on agar plates, Western blot using the BbAFP1 antibody indicated that the BbAFP1 formed a distinct halo around the wild-type colony that was lost for the **Δ***BbAFP1* mutant and noticeably larger for the *BbAFP1*^*OE*^ strain (S13A Fig). BbAFP1 was also detected in protein extracts from agar inoculated with wild type or *BbAFP1*^*OE*^ strains (S13B Fig).

**Fig 9:**
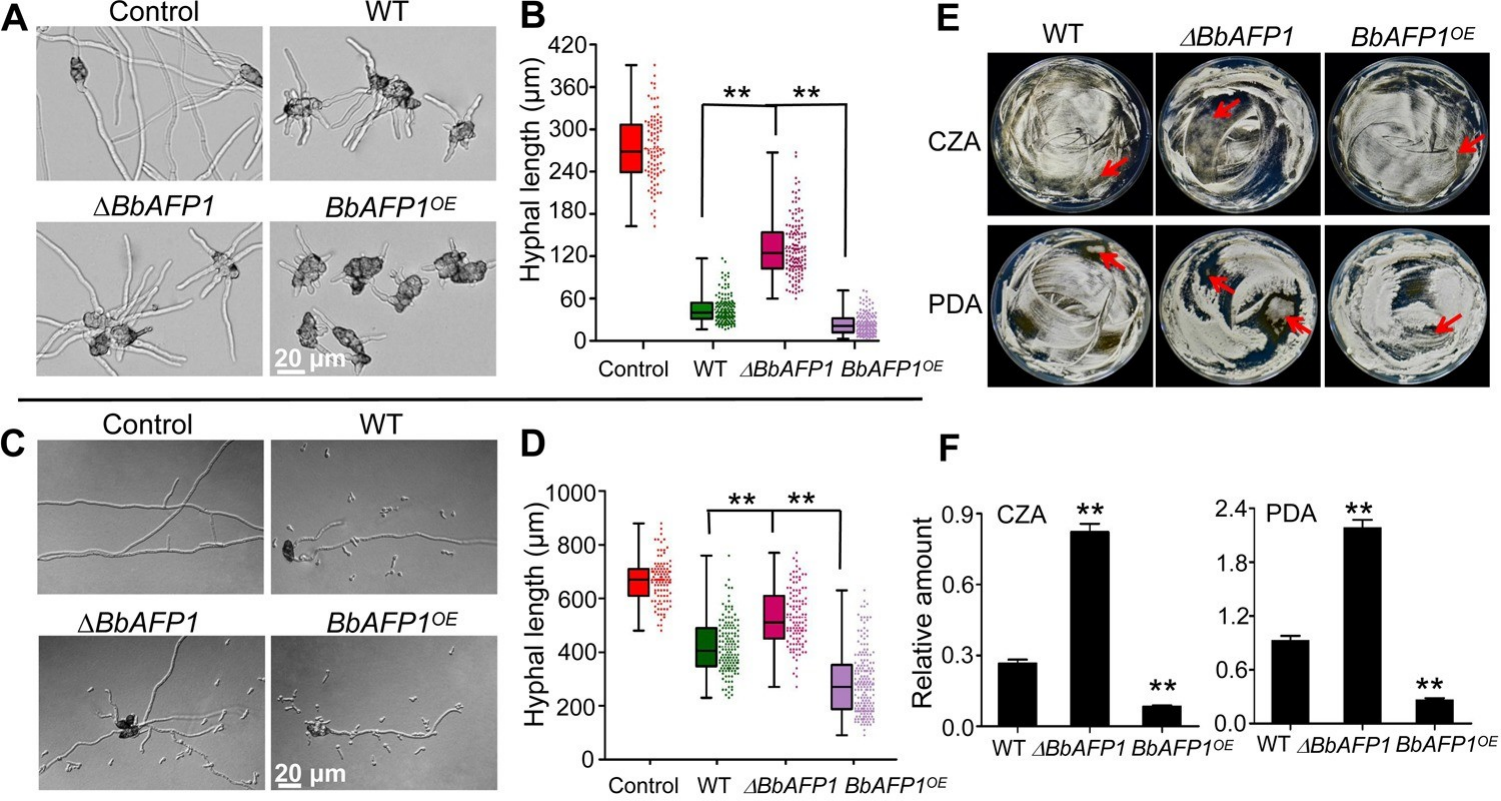
Consequence of *BbAFP1* targeted gene deletion and overexpression on *A*. *brassicae* antifungal activity. (A, B) Representative images and quantification of fungal growth inhibition using protein fractions released from conidia (ultrasonic treatment) of *B*. *bassiana* wild type, **Δ***BbAF1* an*d BbAFP1*^*OE*^ as evaluated against *A*. *brassicae* growth in PDB. (C, D) *B*. *bassiana-A*. *brassicae* co-culture experiments. Representative images and quantification of the effects of *B*. *bassiana* wild type, **Δ***BbAFP1* an*d BbAFP1*^*OE*^ on conidial germination of *A*. *brassicae* on CZA using a *B*. *bassiana* test strain:*A*. *brassicae* initial conidial ratio = 30:1. Growth inhibition was evaluated after 14 h of co-cultivation. (E) *B*. *bassiana* test strain-*A*. *brassicae* co-cultivation using an initial conidial ratio = 30:1 and plated on CZA or PDA, 4 d post-coinoculation. Red arrows indicate brown *A*. *brassicae* colonies, with *B*. *bassiana* growth forming white mycelia. (F) Quantification of *A*. *brassicae* growth in co-cultivation as measured by real-time PCR using total genomic DNA extracted from the fungal mixtures on plates. The reference gene used was *actin* (the designed primer was non-specific capable of giving product for both *B*. *bassiana* and *A*. *brassicae*), and the *AbreAtr1* gene of *A*. *brassicae* was used as target gene, with primers designed to amplify the sequence from *A*. *brassicae* but not *B*. *bassiana*. All experiments were performed in triplicate with at least three independent biological samples. Error bars = SD, ** indicates P < 0.01, t-test.

**Fig 10:**
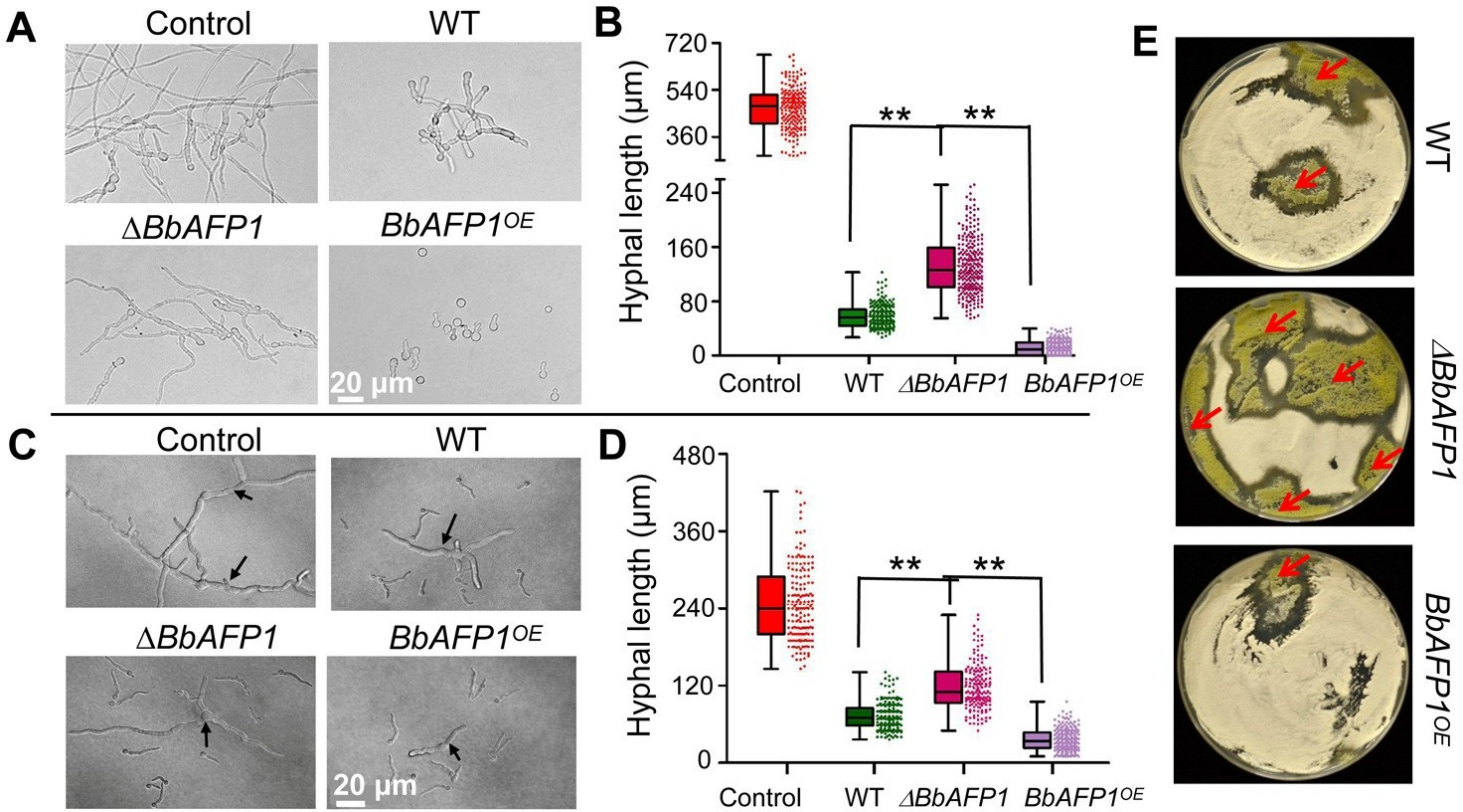
Consequence of *BbAFP1* targeted gene deletion and overexpression on *Trichoderma* sp. antifungal activity. (A, B) Representative images and quantification of fungal growth inhibition using protein fractions released from conidia (ultrasonic treatment) of *B*. *bassiana* wild type, **Δ***BbAF1P* an*d BbAFP1*^*OE*^ as evaluated against *Trichoderma* sp. growth in PDB, 28 h post-treatment. (C, D) *B*. *bassiana-Trichoderma* sp. co-culture experiments. Representative images and quantification of *B*. *bassiana* wild type, **Δ***BbAF1* an*d BbAFP1*^*OE*^ on conidial germination of *Trichoderma* sp. on CZA using a *B*. *bassiana* test strain:*Trichoderma* sp. initial conidial ratio = 10:1. Growth inhibition was evaluated after 17 h of co-cultivation. Black arrows indicate *Trichoderma* sp. (E) *B*. *bassiana* test strain-*Trichoderma* sp co-cultivation using an initial conidial ratio = 10:1 and plated on CZA 4 d post-coinoculation. Red arrows indicate *Trichoderma* sp. colonies. All experiments were performed in triplicate with at least three independent biological samples. Error bars = SD, ** indicates P < 0.01, t-test.

No obvious differences in growth of the wild type, **Δ***BbAFP1*, and *BbAFP1*^*OE*^ strains were seen on 0.5 × SDAY, PDA, or CZA or in terms of the hyphal growth, development, and conidial germination of the three strains (S14A and S14B Fig). Insect bioassays using *G*. *mellonella* as the host indicated no significant differences in virulence between the three *B*. *bassiana* strains (S14C Fig). Co-inoculation assays did not indicate any significantly noticeable differences in terms of the ability of the wild type, **Δ***BbAFP1*, and *BbAFP1*^*OE*^ strains to inhibit/antagonize the growth of *A*. *brassicae*, *B*. *cinerea*, or *Trichoderma* sp. in these types of plate assays (S14D Fig). However, when cell-wall proteins derived from the wild type, **Δ***BbAFP1*, and *BbAFP1*^*OE*^ strains were used to examine (growth) inhibition of *A*. *brassicae* and *Trichoderma* sp, significant differences were seen (Figs 9 and 10). *A*. *brassicae* hyphal length was reduced 6-7-fold using *B*. *bassiana* wild type cell-wall proteins as compared to untreated controls (Fig 9A and 9B). Treatment with the **Δ***BbAFP1*-derived cell-wall proteins resulted in ~4-6-fold reduction in *A*. *brassicae* as compared to untreated cells, which was statistically different from the wild type effect (~2.5-fold decrease, P < 0.05). In addition, *BbAFP1*^*OE*^-derived cell-wall proteins resulted in 20-25-fold overall inhibition of *A*. *brassicae* growth that was significantly different from the wild type and **Δ***BbAFP1*-derived cell-wall proteins (P < 0.01 and P < 0.01, respectively). A similar pattern of inhibition of *Trichoderma* sp. was seen using the various *B*. *bassiana* derived cell-wall proteins (Fig 10A and 10B). In a more qualitative competition assays, fungal spores of the wild type, **Δ***BbAFP1*, and *BbAFP1*^*OE*^ *B*. *bassiana* strains were mixed with *A*. *brassicae* or *Trichoderma* sp. (30:1 and 10:1, respectively) and plated onto CZA. For *B*. *bassiana*-*A*. *brassicae* mixture, enhanced growth of the competing *A*. *brassicae* was apparent for **Δ***BbAFP1-A*. *brassicae* mixture (25% increase in hypha length, P < 0.01), whereas reduced growth of *A*. *brassicae* was seen for the *BbAFP1*^*OE*^*-A*. *brassicae* mixture (30% decrease, P < 0.01) as compared to the wild type-*A*. *brassicae* combination (Fig 9C and 9D). After further growth of the plates up to 4 days, more and bigger colonies of *A*. *brassica* were appeared in the **Δ***BbAFP1-A*. *brassicae* plates compared to *BbAFP1*^*OE*^/wild type-*A*. *brassicae* plates (Fig 9E). RT-PCR analysis with total genomic DNA of mixture as template indicated BbAFP1-producing strains decreased the relative abundance of *A*. *bassicae* by ~3-fold (wild type) and 8-fold (*BbAFP1*^*OE*^) compared to BbAFP1-deleted strain (Fig 9F). More obvious inhibition effects on hyphal growth and colony formation of *Trichoderma* sp. were also observed in the presence of *B*. *bassiana* wild type and *BbAFP1*^*OE*^ compared to **Δ***BbAFP1* (Fig 10C–10E).

### Expression of *BbAFP1* in tomato confers resistance to plant pathogenic fungi

**Fig 11:**
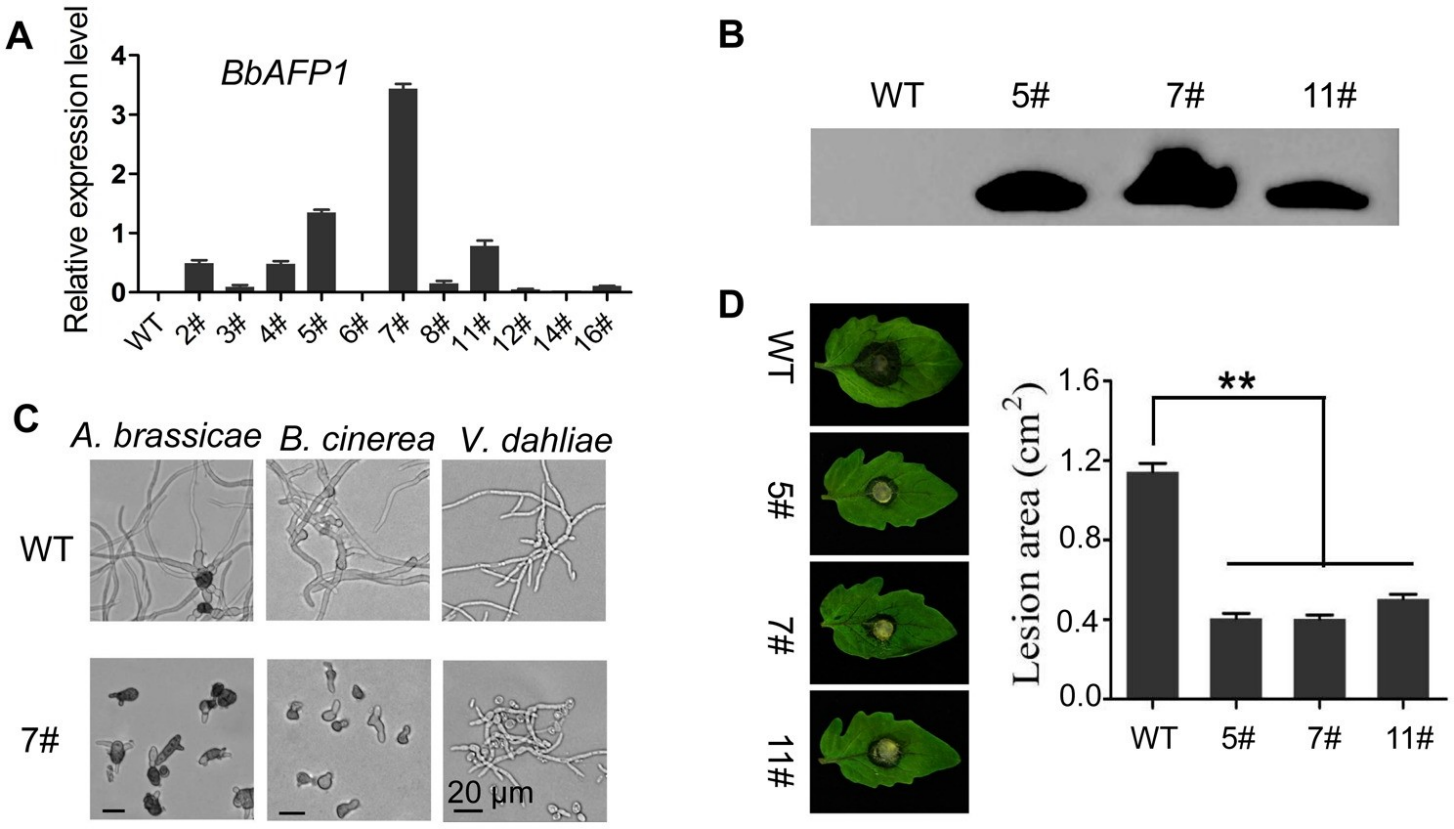
Transgenic expression of *BbAFP1* in tomato (*S*. *lycopersicum*) and effect on resistance to fungal pathogens. (A) Expression of *BbAFP1* in independent transgenic tomato lines as examined by qRT-PCR with tomato *tublin* as the reference gene. (B) Western blot analysis of BbAFP1 protein production in transgenic tomato lines. Anti-BbAFP1 antibody was used as primary antibody. (C) Inhibition of *A*. *brassicae* (representative image 12 h post-treatment), *B*. *cinerea* (15 h post-treatment), and *V*. *dahliae* (24 h post-treatment) by leaf extracts of BbAFP1-expressing transgenic tomato. (D) Detached leaf bioassays of disease progression in plants inoculated with *B*. *cinerea*. Lesion area caused by *B*. *cinerea* was measured. Representative images were taken 3 d post inoculation, respectively. WT, wild-type tomato; 5#/7#/11#, transgenic tomato lines. Error bars = SD, ** indicates P < 0.01, t-test.

As BbAFP1 showed inhibitory activity against phytopathogenic fungi *in vitro*, we were interested in whether this gene could confer protection from fungal infection *in planta*. To test this, BbAFP1 containing its native signal peptide was introduced into tomato (*Solanum lycopersicum*) by *Agrobacterium tumefaciens*-mediated transformation as detailed in the Methods section. After selected on kanamycin-contained medium, the obtained transformants were further confirmed by PCR, RT-PCR and Western blot and several transgenic lines (5#, 7#, and 11#) with high-level expression of BbAFP1 were selected for disease resistance analysis (Fig 11A and 11B). The transgenic plants and wild type showed similar morphology, with no obvious difference detected in overall growth or morphology (S15 Fig). Leaf extracts of transgenic plants showed significant inhibitory activity against conidial germination and hyphae growth of *A*. *brassicae*, *B*. *cinerea*, and *V*. *dahliae* compared to that of wild type (Fig 11C). *B*. *cinerea* detached leaf inoculation bioassays were used to test *in planta* resistance to a plant fungal pathogen. At 3 d post-inoculation of *B*. *cinerea*, BbAFP1-expressing tomato plants showed significantly lowered disease symptoms as compared to wild type plants (Fig 11D). The lesion area caused by *B*. *cinerea* was 3-fold larger in wild type than that in BbAFP1-expressing plants (1.2 cm^2^ vs 0.4 cm^2^, P < 0.01) (Fig 11D).

## Discussion

Based on the database of antimicrobial peptides at http://aps.unmc.edu/AP/main.php, approximately 3072 antimicrobial peptides have been registered, but only 20 AMPs are from fungi. With the availability of fungal genomic data, more putative AMPs have been found [41, 42]. Of these, only a handful have been characterized mainly in terms of structure and antifungal activity; to date the mechanism(s) of their biological functions in fungi have remained obscure. In the present study, we identified an antifungal peptide BbAFP1 from *B*. *bassiana*, which also showed effective antifungal activity against select filamentous fungi. Addition of BbAFP1 to susceptible fungi resulted in the induction of significant aberrant morphological changes as has been reported to a variety of other fungal AFPs [43, 44]. Amino acid comparison to previously characterized fungal AFPs indicated a unique distribution of charged residues and a relatively high theoretical pI (9.01) for the mature protein. Phylogenetic analyses indicated the distribution of fungal AFPs into at least 3–4 broad clades [11, 19], with the *B*. *bassiana* protein belongs to PAF class.

Substrate binding analyses showed that BbAFP1 possessed the ability to bind to chitin and zymosan with little to no binding to chitosan or cellulose. A phenylalanine (F^50^) was identified as critical for chitin binding and shown to contribute to antifungal activity, with mutation of this residue to alanine reducing both activities. This finding is consistent with the ability of some AFPs can bind chitin or be purified via chitin affinity chromatography [45, 46] and the identification of a putative chitin binding domain, similar to the bacterial type-3 chitin binding domain, within which the F^50^ residue is right in the middle (i.e. CKFKGQ, Meyer 2008) [44]. The chitin binding has been suggested to be related to at least one proposed mechanism of AFP functioning, namely by inhibiting chitin synthases [30], however, BbAFP1 had no effect on chitin synthesis in *A*. *brassicae* or *F*. *graminearum*. In addition, the F^50^ mutant retained full activity towards the oomycete, *P*. *nicotianae*. A simple explanation for these results is that antifungal activity is enhanced by the chitin binding, whereas anti-oomycetal activity remains unaffected due to the lack of chitin in the cell walls of the latter organism. Aside from its ability to bind chitin, our data show that BbAFP1 also displays affinity to zymosan (which contains both chitin and glucans) as well as directly to glucan particles. These data suggest that AFPs may possess alternate (to chitin) binding targets in target cells, and would be consistent with the anti-oomycetal activity of these peptides. The existence of multiple binding targets in cell walls would act to (1) increase ability to be retained on fungal cell walls, and (2) expand the range of these peptide to non-fungal organisms and/or to fungal cells that may be able to mask their chitin (or vice versa). Treatment of *F*. *graminearum* with nikkomycin, a chitin synthase inhibitor, led to a significant upregulation of the expression of genes involved in chitin and glucan synthesis. These findings are consistent with well described compensatory mechanisms, i.e. effects on one component of the fungal cell-wall, alters the expression of biosynthetic genes involved not only in the original component but that of other components as well. In contrast, BbAFP1 does not appear to alter expression of either chitin or glucan biosynthetic genes. These results suggest that BbAFP1 has a multipronged binding targets in host cell walls, with binding (ability) independent of the antibiotic activity. Thus, the cell wall affinities of BbAFP1 likely acts to improve targeting and delivery. Intriguingly, BbAFP1 could also bind to the conidial cell wall of *B*. *bassiana* itself but was unable to bind to germinated conidia (neither at the conidial base nor on the growing hyphae), suggesting a mechanism for self-protection by blocking access of the peptide to the cell wall of the growing fungus.

Disruption of target membrane integrity by AFPs is considered the main mechanism by which these peptides inhibit fungal growth, however the nature of this interaction remains obscure. The *P*. *chrysogenum* PAF protein has been suggested to function at least in part via membrane hyperpolarization and induction of apoptotic pathways in susceptible hosts via activation of specific G-protein signaling pathways [29, 43] although how the peptide induces these downstream pathways is unknown. The membrane sphingolipid glucosylceramide (GlcCer) in *Neuospora crassa* has been shown to play an important role in fungal susceptibility toward the *P*. *chrysogenum* PAF peptide [47], with deletion of genes involved in GlcCer biosynthesis resulting in increased resistance against PAF [47]. In the present work, we show that the mechanism of BbAFP1 activity involves disruption of host target membrane potential change, with a resultant ROS burst inside the cell, resulting in membrane disruption, leakage, and ultimately in cell death. In terms of structural features, the γ-core motif composed of a conserved amino acid sequence [GXC]-[X_3−9_]-[C] that has been identified in cysteine-rich antimicrobial proteins as well as in AFPs of ascomycetes has been implicated in exerting direct antimicrobial activity [11, 48]. Peptides, spanning the γ-core motif, and consisting of a 14-aa stretch (termed Pγ) from PAF with an increased overall net positive charge and increased hydrophilicity displayed a 10-fold increase in antifungal activity when compared to the native sequence [11]. In addition, the γ-core motif appears to be directly involved in mediating protein-membrane interaction [49], and a conserved γ-core motif (GICTKAKNEC) can also be found in BbAFP1. Within the (protein) mutational analyses conducted in this study, the sites selected in BbAFP1 for chitin binding-antifungal activity analyses included Y^37A^, F^50A^, F^59A^, Y^74A^, and Y^79A^, all of which are located outside of the γ-core motif. Except for BbAFP1^F50A^ which showed decreased chitin binding and antifungal activity, the other mutations have little effect on antifungal activity. Intriguingly, the putative chitin binding domain CKFKGQ (48–53) is adjacent (downstream) to the γ-core motif (39–48), suggesting that the region in BbAFP1 spanning from C^39^ to Q^53^ may represent a core sequence involved in antifungal activity and chitin binding. However, the fact that the BbAFP1^F50A^ mutant which lies just outside the γ-core (but in the putative chitin binding domain) results in loss of both chitin binding and antifungal activity, first provides evidence for verification of the chitin binding domain, but also suggests that sequences outside of the γ-core are important for antifungal activity. Our data concerning the action mechanism of BbAFP1 (i.e. disruption of cell membrane integrity, elicitation of a ROS burst, and cell death), is consistent with what has been reported for the *Penicillium chrysogenum* PAF targeting sensitive *Aspergillus* strains, and the *Heuchera sanguinea* HsAFP and the human salivary peptide histatin 5 targeting *Candida albican* [50–52]. In addition, arenicin-1, from the marine polychaeta lugworm (*Arenicola marina*) has been shown to induce ROS production in *C*. *albicans*, and human cathelicidin and melittin (from bees) have both been shown to result in the production of oxidative species in target cells [53–55].

Within broader environmental and (fungal) lifestyle aspects, to date, the biological context of how fungal AFP function has not yet been reported. Indeed, in several instances, the very conditions under which fungal AFPs are produced are unknown. Although gene expression studies indicated expression of an AfpB in the citrus pathogen *P*. *digitatum*, including during infection, the protein itself could not be detected in any strains or growth conditions examined [19]. Of three putative AFPs identified in *P*. *expansum*, only PeAfpA was found in fungal culture supernatants, with the protein showing broad spectrum antifungal activity against filamentous fungi and yeasts [18]. Heterologous expression analyses indicated that PeAfpB was effective against a more restricted range of filamentous fungi but was inactive against yeasts (similar to our results with BbAFP1), whereas PeAfpC was largely ineffective against the different fungi tested [18]. Gene expression studies on the *P*. *chrysogenum paf* gene, have indicated some induction under various stress conditions, but not during co-cultivation experiments and correlation to actual protein production is lacking in many circumstances [13, 21, 56]. In addition, another function for the PAF protein has been suggested where it acts to enhance conidiation, with deletion mutants of *paf* producing reduced conidial production by ~2-fold [43]. More recently, AFPs have been suggested as acting as cannibal toxins with primary function to kill genetically identical siblings [1].

Our data indicate a more straight-forward, albeit nuanced biological context for the functioning of (at least some) AFPs. BbAFP1 appeared to be exclusively expressed in aerial conidia and not during any other growth stages or differentiated cell type, i.e. submerged conidia or blastospores, nor was the protein expressed in the presence of competing fungi or during infection of target insect hosts except on new conidia formed at the last stage of the development. Through analysis the promoter region, a binding site (CATTCT) for transcription factor AbaA, which regulates conidial development in fungi [57], was observed, further indicating the production of BbAFP1 was related to conidiation. BbAFP1 was localized to the cell walls of conidia, where it was released into the surrounding environment within 4–6 h after the germination process had begun. Note that this is largely before actual germ tubes are apparent (~6–8 h), with no production in germlings, or growing hyphae/mycelia until new spores are produced (~6–10 d into the growth cycle). The early time point of release of BbAFP1 into the surrounding environment supports this as a critical, i.e. vulnerable, phase for resting spores as they begin to seek to acquire nutrients in a potentially hostile environment. Thus, we propose that BbAFP1 acts as a competition factor that is “pre-loaded” into spore cell walls and released to minimize the activities of competing fungal microbes. The competitive advantages of such a system are apparent. Deletion mutants of *BbAFP1* or strains engineered to constitutively-express the peptide were largely unaffected in fungal morphology or development (unlike what has been reported for *P*. *chrysogenum* paf [43]), but the deletion mutant was at a disadvantage whereas the over-expressing strain was better capable of competing in co-cultivation experiments with various other filamentous fungi. Thus, particularly within the context of soil and plant-root and phylloplane environments the production of BbAFP1 would provide a distinct advantage. In addition, our data provide one mechanism by which so-called “beneficial” microbes may function to enhance and/or protect plants from plant pathogens. By releasing the peptide into the environment as the conidia germinates, the fungus would be able to out-compete others in colonization and/or establishment within soils and/or on plants which can include root systems or the stem/leaves of the plant. Intriguingly, we noticed that the conidia-released proteins from BbAFP1 deletion strain also showed antifungal activity although it was lower than that of wild-type or overexpression strain. These data suggest that additional “competition” factors are likely produced by *B*. *bassiana*.

The potential biotechnological applications of AFPs as antifungal agents has been recognized [44, 58]. Topical application of the *A*. *giganteus* AFP to rice leaves conferred protection *M*. *grisea* infection and application to tomato roots decreased infection by *F*. *oxysporum* [46, 59]. The *A*. *giganteus* AFP has also been transgenically expressed in wheat and rice, conferring protection against a variety of filamentous fungi [60, 61]. It is interesting to note that this AFP belongs to a different clade/class than the *B*. *bassiana* protein and that the native signal peptide was replaced with a plant signal peptide for expression in the target plants. Here, we show that transgenic expression of BbAFP1 with its native peptide in tomato results in enhanced resistance of tomato against infection by *B*. *cinerea* in detached leaf assays. Overall, our results provide the functional biological context of BbAFP1 activity in the fungus and expand the relevance of AFPs as antifungal agents capable of increasing crop resistance to fungal plant pathogens, particularly as this class of AFPs appears to have little to no effects outside of target filamentous fungi, i.e. no discernable bacterial, plant, or higher eukaryote (animal) toxicity [62–64].

## Materials and methods

### Strains and media

*B*. *bassiana* CGMCC7.34 (China General Microbiological Culture Collection Center) was conserved as a mixture of dry conidia at −80°C. Fungal strains were routinely grown in/on potato dextrose broth/agar (PDB/PDA), Sabouraud dextrose broth/agar (SDB/SDA) or Czaped-Dox broth/agar (CZB/CZA). *E*. *coli* DH5α was used for gene cloning and vector construction. *P*. *pastoris* GS115 was used for heterologous expression of the antifungal peptide. Fungal strains, including *A*. *brassicae*, *A*. *solani*, *V*. *dahliae*, *F*. *oxysporium*, *M*. *oryzae*, *F*. *graminearum*, *Trichoderma* sp. and *S*. *cerevisiae* were routinely grown on PDA or YPDA (yeast).

### Protein expression, purification, and antibody production

For heterologous expression of BbAFP1 (EJP62050.1), the coding sequence of BbAFP1 without its signal peptide (19 a.a. at the N-terminus, as predicted with SignalP 4.0) was amplified by PCR with the primer pair P1/P2 (S2 Table) using *B*. *bassiana* conidial cDNA as the template and cloned into the pEASY-Blunt vector (Transgenic Gen Biotech, China). *BbAFP1* fragment released from pEASY-*BbAFP1* by *Eco*RI/*Not*I was cloned into pPIC9K for transformation and expression in *P*. *pastoris*. To facilitate purification, a 6 × His tag was fused at the C-terminus of BbAFP1 (primer P2). *P*. *pastoris* transformation and colony screening were performed according to the manufacturer’s recommendations (Invitrogen, USA). For expression of BbAFP1 in *P*. *pastoris*, clones were initially cultured in BMGY (Buffered Glycerol-complex Medium) and then induced in BMMY (Buffered Methanol-complex Medium) with 0.5% methanol (v/v) added daily for up to 4 d. The supernatant derived from *P*. *pastoris* cultures was concentrated using PEG 20, 000 in a dialysis bag with a molecular weight cut-off (MWCO) of 1000. The concentrated sample was then subjected to desalting chromatography (GE Healthcare Life) with phosphate-buffered saline (PBS, 50 mM, pH 7.0) as the mobile phase. The BbAFP1 fraction was then purified by His-tag affinity chromatography (Invitrogen, China), eluting at an imidazole concentration of 500 mM. The protein samples were treated with an additional desalting chromatography step. Concentrations of the purified BbAFP1 was normalized by the Bradford method using bovine serum albumin (BSA) as the standard curve. Polyclonal antibodies to BbAFP1 were produced in rabbit using the purified protein (Genscript, China). Antibodies were purified using Protein A column. BbAFP1 production in *P*. *pastoris* was analyzed by sodium dodecyl sulfate-polyacrylamide gel electrophoresis (SDS-PAGE) and Western blot using antibodies against BbAFP1 as indicated.

### Expression and localization analysis of BbAFP1

The *BbAFP1* ORF with its native promoter (*BbAFP1*_*promoter*_::*BbAFP1*, ~1.3 kb) and sole promoter region (*BbAFP1*_*promoter*_, ~1.0 kb) were amplified by polymerase chain reaction (PCR) with primer pair Ppafpp1/Ppafpp3 and Ppafpp1/Ppafpp2, respectively, and the open reading frame of (enhanced) green fluorescent protein (*eGFP*) was PCR amplified with primer pair Pegfp1/Pegfp2. The obtained *BbAFP1*_*promoter*_ and *eGFP* nucleotide fragments were cloned into the *Hin*dIII site of pK2-*bar* (containing the *bar* marker conferring resistance to phosphinothricin) by seamless cloning (ClonExpress MultiS One Step Cloning Kit, Vazyme, China) to yield pK2-*bar*-*BbAFP1*_*promoter*_::*eGFP*. Similarly, *BbAFP1*_*promoter*_::*BbAFP1* and *eGFP* were cloned into the pK2-*bar* to yield pK2-*bar*-*BbAFP1*_*promoter*_::*BbAFP1*::*eGFP*. The former vector was used for expression analysis of BbAFP1 and the latter for localization analysis. The integrity of the constructs was verified by sequencing (Invitrogen, China) and the plasmid was transformed into *Agrobacterium tumefaciens* AGL-1. *A*. *tumefaciens* mediated transformation of *B*. *bassiana* was performed as described [65]. eGFP expression in the reporter strain (*BbAFP1*_*promoter*_::*eGFP*) during various fungal developmental stages was examined by laser confocal microscopy (FV1000, Olympus) after culturing on CZA over the indicated time course. Gene expression analyses of *BbAFP1* was monitored by real-time quantitative PCR using primers PBbAFPrt-1 and PBbAFPrt-2 as described [66]. Total RNA was isolated from different fungal developmental stages using EASYspin rapid extraction kit (Biomed, China) and converted to cDNA using HiScript II Q RT SuperMix for qPCR kit (Vazyme, China). Fungal co-culture experiments using the *BbAFP1*_*promoter*_::*eGFP* reporter systems were performed on PDA plates. For experiments in liquid media, *B*. *bassiana* and test fungi (*A*. *brassicae*, *V*. *dahliae*, *B*. *cinerea*) were pre-cultured in PDB for 2 d (200 rpm, 26°C), then mixed *B*. *bassiana* and test fungus together and cultured for additional 12–48 h. Aliquots of cells were removed and directly visualized using both differential contrast (DIC) and confocal microscopy. For agar plate, *BbAFP1*_*promoter*_::*eGFP* strain was inoculated into the edge of test fungal colonies of *A*. *brassicae*, *B*. *cinerea*, and *V*. *dahliae* on PDA and cultured for 3 d, fluorescence in *B*. *bassiana* hyphae was detected. Control cultures were grown in the absence of competing fungi. For experiments using the *B*. *bassiana* (*BbAFP1*_*promoter*_::*eGFP*) promoter reporter strain during infection, conidial suspensions (2 × 10^7^ conidia/ml) were topically inoculated onto *Galleria mellonella* larvae and eGFP signal was examined in various cell type during the infection course (4 h-4 d) and in hyphae and conidia on/in cadaver post death (6 d). *In vivo* hyphal bodies derived from host hemocoel were obtained as described [67]. The wild type *B*. *bassiana* strain was used as a negative (fluorescent microscopy) control and a *B*. *bassiana* transformant containing the constitutive eGFP construct (*PgpdA*::*eGFP*), in which the *eGFP* ORF was placed under control of the constitutive *B*. *bassiana* glyceraldehyde-3-phosphate dehydrogenase promoter (*PgpdA*) was used as a positive control. The expression and localization of BbAFP1::eGFP was observed during various fungal growth and developmental stages. For some experiments, cells were co-stained with the vesicle membrane dye FM4-64 as described [68].

### Construction of targeted gene knockout and overexpression strains

For construction of the *BbAFP1* gene deletion vector, the phosphinothricin actetyltransferase resistance gene (*bar*) cassette (~1.6 kb) was used to replace a 440 bp gene fragment containing 238 bp of the 5’-terminus of *BbAFP1* gene. The upstream and downstream fragments of the construct were amplified via PCR with primer pairs PafpLB1/PafpLB2 and PafpRB1/PafpRB2, respectively, using *B*. *bassiana* genomic DNA as the template (S2 Table) and cloned into the plasmid vector pK2-*bar*. Putative homologous recombination transformants corresponding to *ΔBbAFP1* were screened with primers PBbafpt1 and PBbafpt2 for the correct integration event using a simple template preparing method essentially as described [66]. For construction of the *BbAFP1* overexpression vector, the ORF of *BbAFP*1 and *BbgpdA* promoter fragment were amplified with POEAFP-1/POEAFP-2 and PgpdA-1/PgpdA-2, respectively. The obtained *BbAFP1* and *BbgpdA* fragments were cloned into the *Hin*dIII site of pK2-*bar* vector by seamless cloning. *BbAFP1* overexpression transformants *BbAFP1*^*OE*^ were obtained by *A*. *tumefaciens* mediated transformation and transformants with high-level expression of *BbAFP1* were screened by quantitative reverse transcriptase-(RT)-PCR. Briefly, 7 transformants and wild type were grown on 30 ml of PDB media for 3 d, before harvesting of the fungal cells. Total RNA extraction and RT-PCR analysis were described above (Expression and localization analysis of BbAFP1 section).

### BbAFP1 release from conidial cell walls

BbAFP1 releases from fungal cells was examined using conidia derived from the *BbAFP1*_*promoter*_::*BbAFP1*::*eGFP* collected from CZA (14 d) in 0.05% Tween-80. Conidial suspension (600 μl, 3 × 10^8^ conidia/ml) in 1.5 ml Eppendorf tube was treated by sonication (40 KHz) for 10 min. After centrifugation at 10, 000g for 10 min, the fluorescence signal in the supernatant was measured by microplate reader (infinite M200 PRO, TECAN) and residual fluorescence in conidia were observed by confocal microscopy (FV1000, Olympus) excited with a 488 nm line of the argon laser. To precipitate proteins in the supernatant, 150 μl of trichloroacetic acid (TCA) was added to 600 μl supernatant and the mixture was placed on ice for 15 min. After centrifugation at 12, 000g for 10min, the pellet was washed with 200 μl ice-cold acetone five times and the pellet air dried. The pellet was dissolved in 150 μl distilled water and subjected to Western blot probed with an anti-GFP antibody (Genescript, China).

Release of BbAFP1 into the surrounding media was also examined in both liquid and solid media (CZB and CZA). In liquid media, conidial suspension of *BbAFP1*_*promoter*_::*BbAFP1*::*eGFP* or wild-type was added into 15 ml CZB (final concentration = 2–20 × 10^6^ conidia/ml) and cultured at 26℃ for 15 h. Aliquots of cells were removed every 3 hrs and the cell-free culture supernatants were obtained by centrifugation (10, 000g, 10 min, 25°C). The fluorescence intensity resulting from the protein product of the *BbAFP1*_*promoter*_::*BbAFP1*::*eGFP* gene cassette was detected (infinite M200 PRO, TECAN). In addition, BbAFP1 released into the media was quantified by ELISA using the generated anti-BbAFP1 antibody. Proteins fractions derived from cell-free culture supernatants were coated onto 96-well plates for 2 h at 37°C then ELISA analysis was performed as described [69], and purified BbAFP1 from *P*. *pastoris* was used to prepare a standard curve. In solid media, fluorescence detection and Western blot were used to detect the release of BbAFP1. First, 100 μl of a conidial suspension (1 × 10^7^ conidia/ml) was spread onto CZA plates and cultured at 26°C for 12 h. Agar sections (1 cm × 1 cm) were cut from plates and conidia on the agar directly subjected to fluorescent signal detection every two hours. Second, conidial suspension (5 μl, 3 × 10^8^ conidia/ml) was inocualted onto CZA plates and incubated at 26℃ for 16 h. The agar in the plate was carefully removed and proteins present in the agar media were directly transferred onto PVDF membranes by electroblotting and the membrane probed using the anti-BbAFP1 antibody and goat anti-rabbit lgG (Thermo, USA) as the secondary antibody using standard procedures. BbAFP1 was also detected in protein extracts from agar incubated with *B*. *bassiana* strains by Western blotting. Briefly, the agar around the colony was cut and placed into a 0.5 ml Eppendorf tube which was perforated at the bottom by using a syringe needle and the holes were blocked with glass cotton. After frozen in liquid nitrogen for 30 s, the 0.5 ml Eppendorf tube was placed into a 1.5 ml Eppendorf tube and centrifugated at 12, 000g for 2 min. Protein solution in 1.5 ml Eppendorf tube was precipitated with TCA as described above.

### Antimicrobial assays

***B*. *bassiana* conidial cell wall extract.** The inhibitory activity of BbAFP1 was analyzed versus *A*. *brassica* visually as *A*. *brassica* conidia are morphologically distinct from *B*. *bassiana* cells and can be easily discerned. In the first treatment, protein fractions were prepared from 500 μl conidial suspensions (3 × 10^8^ conidia/ml) of test strains grown on CZA plates (14-d old) and harvested into 0.05% Tween-80 by sonication of the cells as described above. The cell-free protein extract supernatant was obtained by centrifugation (12, 000 rpm, 10 min). A mixture containing 10 μl cell-free conidial sonication protein extract supernatant, 1 μl *A*. *brassica* conidial suspension (1 × 10^6^ conidia/ml) and 4 μl PDB was spotted onto a glass slide and placed in a 120-mm plate with wet filter paper to keep moisture. After culturing at 26℃ for 10 h, the *A*. *brassica* growth was observed microscopically and germination and hyphal length was measured with Image J soft (n > 150). In the second treatment, 30 μl *B*. *bassiana* conidia (6 × 10^7^ conidia/ml) derived from the various test strains were mixed with 30 μl *A*. *brassica* conidia (2 × 10^6^ conidia/ml) and spread onto CZA plates. After culturing at 26℃ for 14 h, fungal growth was examined microscopically and germination/hyphal length was measured as described above. Samples were further cultured for up to 4 d and colonies of *B*. *bassiana* and *A*. *brassica* were visually inspected/observed. For *Trichoderma* sp., the same treatment was performed with a conidia ratio = 10:1 (*B*. *bassiana*:*Trichoderma* sp.) as described above (n > 150). Quantification of fungal (*A*. *brassiana*) cell growth was determined by RT-PCR using total genomic DNA isolated from *B*. *bassiana*-*A*. *brassicae* co-cultivation experiments as the template. Actin was used as the reference gene (primer pair; Pactin-m1/2) and was designed based on conserved regions of the gene found in both fungi, i.e. these primers would recognize *actin* gene transcript corresponding to both *B*. *bassiana* and *A*. *brassicae*, and would represent the total amount of fungal growth. The *AbreAtr1* gene (AY246696.1) of *A*. *brassicae*, encoding an ATP-binding cassette transporter was used as the target gene specific for this fungus using primer pairs (PAbreAtr1-1/2) that failed to produce any product from *B*. *bassiana* DNA.

**Purified BbAFP1.** To analyze the antifungal activity of BbAFP1, the purified protein (0–1.5 μg) was added to sterilized filter paper (6 mm in diameter) at sites 10 mm away from the edge of fungal colonies cultured on PDA plates. Colony growth inhibition was observed after placing the plates at 26°C for 2–3 d. Antibacterial activity was analyzed by adding BbAFP1 (0–2 μg) to plates of *E*. *coli*, *E*. *cloacae*, *Pantoea* sp., *Staphylococcus* sp. and *Stenotrophomonas* sp. on LB agar and culturing at 37°C overnight. Inhibition of the growth of *S*. *cerevisiae* was also analyzed on YPDA (yeast extract/peptone/dextrose) plates using the same method used for the bacterial test. NaAC (20 mM, pH 5.4) without protein was used as a control. The effects of BbAFP1 on *A*. *brassicae* conidial germination were also analyzed in PDB. Briefly, a conidial suspension of *A*. *brassicae* (1 × 10^6^ conidia/ml) was inoculated into PDB containing 5.0 μM BbAFP1 and cultured at 26°C for 12–18 h, with germination/growth observed microscopically.

### Characteristics of BbAFP1

To determine the effect of different pH values on the antifungal activity of BbAFP1, analyses were performed in PDB solution at different pH ranges from 3.0–10.0 (pH 3.0, 30 mM citrate-phosphate buffer; pH 4.0 and pH 5.0, 30 mM sodium acetate buffer; pH 6.0–8.0, potassium phosphate buffer; and pH 9.0–10.0, 30 mM carbonate-bicarbonate buffer). *A*. *brassicae* conidia treated at various pH values (in the absence of BbAFP1) were used as a control. To analyze the thermostability of BbAFP1, the protein solution was pretreated at 100°C for 0.5–6 h in a thermal cycler, after which antifungal activity was analyzed in PDB at 26°C (20 mM NaAC, pH 5.4). The relative growth of hyphae was calculated by dividing the hyphal length under various conditions by the hyphal length under standard conditions (in 20 mM NaAC without BbAFP1 at 26°C) followed by multiplication by 100% (n > 150). The minimal inhibitory concentration (MIC) of BbAFP1 against various microorganisms was analyzed as described [7].

### Effects of BbAFP1 on cell membrane integrity of *A*. *brassicae*

*A*. *brassicae* cells used for the following experiments were pre-cultured at 26°C for 5 h in PDB and the concentration of conidial suspension was 1 × 10^6^ conidia/ml except for membrane potential detection and leakage analysis of intracellular contents (1 × 10^7^ conidia/ml). Membrane potential detection using the transmembrane potential-sensitive fluorescent dye DiSC3(5) was performed as described [70] with some modification. The *A*. *brassicae* cells were re-suspended in PBS (20 mM, pH 7.0), then incubated with DiSC3(5) at a final concentration of 30 μM for 1 h at room temperature. After washing 2 times with PBS (20 mM, pH 7.0), BbAFP1 was added to the cells to achieve a final concentration of 5 μM, 10 μM and 20 μM, respectively. Samples were monitored by multi-mode microplate reader (Varioskan LUX, Thermo, USA) with optical excitation and emission wavelengths of 622 nm and 670 nm, respectively. ROS analysis with H_2_DCFDA was performed as described [71] and images were visualized using confocal microscopy (Leica-SP8, Germany) with excitation and emission wavelengths of 488 nm and 535 nm, respectively. To analyze the localization and internalization of BbAFP1 in target fungi, BbAFP1 was labeled with fluorescein isothiocyanate (FITC) as described [72]. After excess FITC was remove by PD MiniTrap^TM^ G-10 (GE, USA), BbAFP1^FITC^ was normalized by the bradford method as aboved. For visualization of the internalization of BbAFP1, *A*. *brassicae* cells were treated with BbAFP1^FITC^ (5 μM), then observed by confocal microscopy at different time points (5 min-60 min). Additionally, double staining with BbAFP1^FITC^ and propidium iodide (PI) was performed to detect the integrity of the plasma membrane. Briefly, *A*. *brassicae* cells were treated with BbAFP1^FITC^ (5 μM) and PI solution (0.05 μg/ml) for 10 min or 60 min, then observed by confocal microscopy (Leica-SP8, Germany). The excitation and emission wavelengths of FITC and PI are 492/520 nm and 536/600 nm, respectively. For BbAFP1^FITC^ and FM4-64 co-localization experiment, *A*. *brassicae* cells cultured at 26°C for 10 h in PDB were then treated with BbAFP1^FITC^ or BbAFP1^F50A_FITC^(2.5 μM) and FM4-64 (1 μM) for 10 min and then observed by confocal microscopy (FV1000, Olympus). FM4–64 and FITC were excited with the 559 nm line of the helium‐neon laser and the 488 nm line of the argon laser, respectively. Wheat germ agglutinin (WGA) lectin was used to stain fungal cell walls as described [67]. The leakage analysis of DNA/RNA from *A*. *brassicae* cells was performed as described [73]. *A*. *brassicae* cells were treated with BbAFP1 (5 μM) at 26°C for 3 h and supernatant was collected by centrifugation at 10, 000g for 10 min. OD_260_ of DNA/RNA in the supernatant was measured in NanoDrop 2000 (Thermo, USA). Proteins in the supernatant were precipitated with TCA as described above and subjected to SDS-PAGE followed by silver staining using Pierce Silver Stain Kit (Thermo, USA).

### Binding of BbAFP1 to substrata

FITC-labeled BbAFP1 were used to assess to binding to chitin and glucan particles (dextran gel, Sephadex G15) following procedures as described [72]. Protein aliquots digested with proteinase K (3 mg/ml) at 50°C overnight were used as the control. Further analysis of binding BbAFP1 to chitin, chitosan, cellulose, and zymosan was performed using a series of elution methods [72]. Briefly, after BbAFP1 was allowed to bind to various test substrates, the material was treated with either 20% NaCl, 6 M urea, or boiled in 2% SDS; each for 5 min. Suspensions were pelleted by centrifugation (12, 000 rpm, 5 min) and the eluted fraction supernatants were subjected to SDS-PAGE analysis.

### Expression level of genes involved in cell wall synthesis and chitin content assay

Test fungi (*A*. *brassicae* and *F*. *graminearum*) were pre-cultured in PDB for 2 d, then took 1 ml to 50 ml PDB containing 5 μM BbAFP1 or 50 μM Nikkomycin (chitin synthesis inhibitor) and cultured for additional 2 d. Total RNA was isolated as above and the expression level of chitin synthase genes (FGSG_10116, FGSG_03418, FGSG_02483, FGSG_10327, FGSG_01272, FGSG_12039, FGSG_01949) or glucan synthesis related genes (FGSG_07946, FGSG_06998) in *F*. *graminearum* were analyzed by real-time quantitative PCR using the primers listed in S2 Table. Chitin contents of *A*. *brassicae* and *F*. *graminearum* were measured as previously described [74]. N-acetylglucosamine was used to prepare the standard curve and chitin content was calculated (μg/per milligram lyophilized mycelium).

### Mutational analysis

As aromatic acids typically play important roles in the chitin-binding ability of proteins [72], amino acids Y^37^, F^50^, F^59^, Y^74^, and Y^79^ were individually mutated to alanine using the primers listed in S2 Table. Briefly, the γ-phosphate of ATP was transferred to the 5’-terminus of the primers by T4 polynucleotide kinase (NEB), reverse PCR was then performed using pEASY-*BbAFP1* (Protein expression, purification, and antibody production section) as the template with each respective primer pairs. The obtained products were self-ligated and transformed into competent *E*. *coli* cells. The integrity of the desired mutations of *BbAFP1* were confirmed by sequencing of the constructs. The BbAFP1 mutants were expressed in *P*. *pastoris* and purified as described above. The antifungal activity and substrata-binding ability of these mutants were analyzed. The relative growth of hyphae was calculated as described in Characteristics of BbAFP1 section (n > 150).

### Expression of BbAFP1 in tomato

To express *BbAFP1* in tomato, the cDNA sequence encoding full length BbAFP1 was amplified with primer pairs PBbAFP-3/-4 and cloned into the pLGN vector with the constitutive CaMV 35S promoter and a terminator sequence derived from the nopaline synthase gene (NOS) [75]. The integrity of the desired construct was confirmed by sequencing and the plasmid was transformed into *A*. *tumefaciens* LBA4404, which was subsequently used for plant transformation performed as described [76] using *S*. *lycopersicum* as the host. The expression of *BbAFP1* in transgenic tomatoes was analyzed by RT-PCR with the tomato *tublin* (S2 Table) as the reference gene, as well as by Western blot using plant extracts. Total proteins of leaf tissue were extracted by grounding 0.2 g fresh leaves in a mortar with 1 ml 50 mM sodium acetate buffer (pH5.4) on ice. After centrifuged at 4°C for 20 min (12, 000 rpm), total protein content in the supernatants was quantified by the Bradford method. Aliquots of the leaf extracts were tested for fungal growth inhibition (final protein concentration 0.6 mg/ml) against *A*. *brassicae*, *B*. *cinerea*, and *V*. *dahliae* as described above. Fungal disease resistance of transgenic plants was assessed using detached leaf bioassays. Briefly, leaves detached from BbAFP1 transformed and control plants were inoculated with *B*. *cinerea*. For *B*. *cinerea* inoculations, a plug (Φ = 0.5 cm) from a fungal colony was placed on the leaves (n = 30) as described [77]. The treated leaves were placed in a plastic basket and covered with transparent plastic film in order to maintain a high relative humidity. After treatment for 3 d, the symptoms of fungi-infected leaves were observed and lesion area was measured.

## Supporting information

S1 Fig Protein sequence analysis of BbAFP1. (A) Phylogenetic tree of BbAFP1 and its homologous proteins. Phylogenetic analysis was performed with MEGA soft and as described by Sonderegger *et al*. (2018). The name of characterized proteins was given in parenthesis. (B) Alignment of protein sequences of BbAFP1, PAF, GAMA and ANAFP by Clustal W method. Identical aa sequences are highlighted in grey, the signal peptide cleavage site is indicated by an arrowhead. The putative prosequence cleavage site of PAF is LDAR (Meyer 2008). A similar site (FEAR) is found in BbAFP1 and marked by four asterisks. In the mature protein region of BbAFP1, aromatic amino acids which are mutated into alanine are indicated with red boxes, Lys and Arg residues are indicated with green fill boxes and conserved Cys residues are indicated with black boxes.
(TIF)

S2 Fig Inhibition activity of BbAFP1 against bacteria and yeast. P, indicated positive control (10 μg cefotaxime sodium for bacteria and 50 μg geneticin for yeast); 1–4, indicated 0, 0.5, 1.0 and 2.0 μg BbAFP1 was added, respectively. The same sequence of samples was used in all plates. Bacteria were cultured at 37℃ overnight on LB plates. *S*. *cerevisiae* (yeast) was cultured at 30℃ for 2 d on YPDA plate.
(TIF)

S3 Fig Effects of BbAFP1 on conidial germination rate. Purified BbAFP1 (5 μM) was added into the conidial suspension of *A*. *brassicae*. 20 mM NaAC (pH 5.4) was used as a control. Conidial germination rates of *A*. *brassicae* were calculated 12 h or 18 h post-inoculation. All experiments were performed in triplicate with at least three independent biological samples. Error bars = SD.
(TIF)

S4 Fig Release of nucleic acid contents (A) and proteins (B) in the supernatant in BbAFP1-treated *A*. *brassicae* cells. After treated with BbAFP1 (5 μM) for 3 h, the OD_260_ of DNA/RNA was determined and proteins were run in SDS-PAGE and detected by silver staining. All experiments were performed in triplicate with at least three independent biological samples. Error bars = SD.
(TIF)

S5 Fig BbAFP1^FITC^ can bind to cell envelope of conidia but not hyphae in *B*. *bassiana*. *B*. *bassiana* conidia were pretreated with BbAFP1^FITC^ in PDB for 3 h and 15h at 26℃, respectively, then PI was added into the conidia suspension to examine membrane integrity.
(TIF)

S6 Fig Binding of BbAFP1^FITC^ and BbAFP1^F50A_FITC^ to chitin. The fluorescence observation (A) and mean fluorescence intensity quantification (B) of FITC on chitin. We quantified the mean fluorescence intensity by ImageJ software and powdered chitin treated with 20 mM potassium phosphate buffer (pH 6.0) was used as a control. Error bars = SD.
(TIF)

S7 Fig Binding ability and inhibitory activity of BbAFP1 and BbAFP1^F50A^ against *P*. *nicotianae*. (A) Localization of BbAFP1^FITC^ and BbAFP1^F50A_FITC^ on *P*. *nicotianae* conidia. (B) The inhibitory activity of BbAFP1 and BbAFP1^F50A^ against *P*. *nicotianae*. BbAFP1/BbAFP1^F50A^ (2 μg) was spotted onto round filter papers near fungal colony. NaAC (20 mM, pH 5.4) was used as a control.
(TIF)

S8 Fig Glucan binding ability of BbAFP1 and its effects on the expression of genes related to glucan synthesis. (A) Binding of BbAFP1^FITC^ to glucan was determined as detailed in the Materials and Methods section. Glucan treated with 20 mM potassium phosphate buffer (pH 6.0) and Protease K treated BbAFP1^FITC^ were used as a control. (B) Effects of BbAFP1 on the expression level of glucan synthesis related genes, 1,3-beta-glucan synthase (GLs) and the glucan synthesis regulatory protein gene (GLsrp) in *F*. *graminearum*. *F*. *graminearum* was treated with BbAFP1 or the chitin synthesis inhibitor nikkomycin for 2 d, after which total RNA was isolated and RT-PCR analysis was performed with β-*tubulin* as the reference gene as detailed in the Materials and Methods section. All experiments were performed in triplicate. Error bars = SD.
(TIF)

S9 Fig Expression of *BbAFP1* in *B*. *bassiana* cannot been induced by other filamentous fungi on PDA or in PDB. On PDA plates, *BbAFP1*_*promoter*_::*eGFP* strain was inoculated near the colony edge of several filamentous fungi, including *A*. *brassicae*, *B*. *cinerea* and *V*. *dahliae* (top panel). For liquid medium, *BbAFP1*_*promoter*_::*eGFP* strain and test fungus were individually pre-cultured in PDB for 2 d, then mixed them together and cultured for additional 24 h. The expression of *BbAFP1* was detected by GFP fluorescent observation.
(TIF)

S10 Fig Expression analysis of *BbAFP1* during pathogenesis. Time course include before *G*. *mellonella* death (BD, ~72 h post infection) and 24–72 h post death (hpd). A strain constitutively expressing eGFP (*PgpdA*::*eGFP*) was used as positive controls.
(TIF)

S11 Fig BbAFP1 is localized in conidial cell wall. *BbAFP1*_*promoter*_::*BbAFP1*::*eGFP* strain was inoculated onto CZA and fluorescent signal was detected during 0.5–8 d.
(TIF)

S12 Fig Screening of *BbAFP1* knockout mutants and overexpression strains. (A) Schematic of construction of *ΔBbAFP1* mutants. (B) Screening and confirmation of *BbAFP1* knockout strains by PCR. Lane M, Marker 15 (Fermentas), lane 1–3, *ΔBbAFP1* mutants, WT, *B*. *bassiana* wild type. (C) Screening of *BbAFP1* overexpressing strains by real-time PCR.
(TIF)

S13 Fig Western blot analysis of BbAFP1 released into CZA medium. (A) BbAFP1 was detected in situ on agar plates. White circles indicate the inoculation area of *B*. *bassiana* conidia. Red arrows indicate the BbAFP1 signal. (B) BbAFP1 was detected in protein extracts from agar. Antibody against BbAFP1 was used.
(TIF)

S14 Fig Effects of BbAFP1 on colony growth, hyphal growth and antagonistic effects against other filamentous fungi. (A) Colony phenotype of various strains. *BbAFP1*^*OE*^, *ΔBbAFP1* and *B*. *bassiana* wild type strains were inoculated on 0.5 × SDAY, PDA, and CZA plates respectively, and the colony phenotype was observed after cultured the plates at 26 ℃ for 6 days. (B) Hyphal morphology was observed after cultured various stains in PDB for 18 h. (C) Bioassay analysis against *G*. *mellonella* larvae. (D) The antagonistic activity of *B*. *bassiana* strains against various fungi (the central colony) were analyzed on PDA.
(TIF)

S15 Fig *BbAFP1* had no negative impact on the growth and development of transgenic tomato. Plant growth, floral development and fruit size were not significantly different between wild-type and *BbAFP1* transgenic tomato. WT, wild-type tomato; 7#, transgenic tomato line.
(TIF)

S1 Table Parameters of putative BbAFP1 mature protein with several identified fungal AFPs. ^a^Putative parameters. The mature protein of BbAFP1 was deduced by compared the amino acid sequence with that of PAF. The parameters of other fungal AFPs were cited from the references.
(DOCX)

S2 Table Primers used in this study. (DOCX)

S1 Video Internalization process of BbAFP1^FITC^ in *A*. *brassicae* cells. This video shows the internalization process of BbAFP1^FITC^ in *A*. *brassicae* cells. The fluorescent signal was enriched on the surfaces of cells in the beginning, subsequently appeared inside the cells and enhanced gradually. Time-lapse images were acquired in 5 min intervals after treated *A*. *brassicae* cells with BbAFP1^FITC^ for 10 min. Movie plays with 24 frames/s.
(MPG)

S2 Video Detection of ROS burst in *A*. *brassicae* cells in the presence of BbAFP1. This video shows a persistent fluorescent signal detection of H_2_DCFDA in *A*. *brassicae* cells in the presence of BbAFP1. The fluorescent signal in BbAFP1-treated *A*. *brassicae* cells was significantly enhanced in a time dependent manner. Time-lapse images were acquired in 15 s intervals after treated *A*. *brassicae* cells with BbAFP1^FITC^ and H_2_DCFDA for 5 min. Movie plays with 24 frames/s.
(MPG)

S3 Video Detection of ROS burst in *A*. *brassicae* cells in the absence of BbAFP1. This video shows a persistent fluorescent signal detection of H_2_DCFDA in *A*. *brassicae* cells in the absence of BbAFP1. Only weak signal was observed in *A*. *brassicae* cells without BbAFP1. Time-lapse images were acquired in 15 s intervals after treated *A*. *brassicae* cells with H_2_DCFDA for 5 min. Movie plays with 24 frames/s.
(MPG)
