# Supplementary figures and images for: Characterization of a fungal competition factor: Production of a conidial cell-wall associated antifungal peptide
Source: PLoS Pathog. 2020 Apr 23;16(4):e1008518. doi: 10.1371/journal.ppat.1008518 (PMC7200012; doi:10.1371/journal.ppat.1008518)

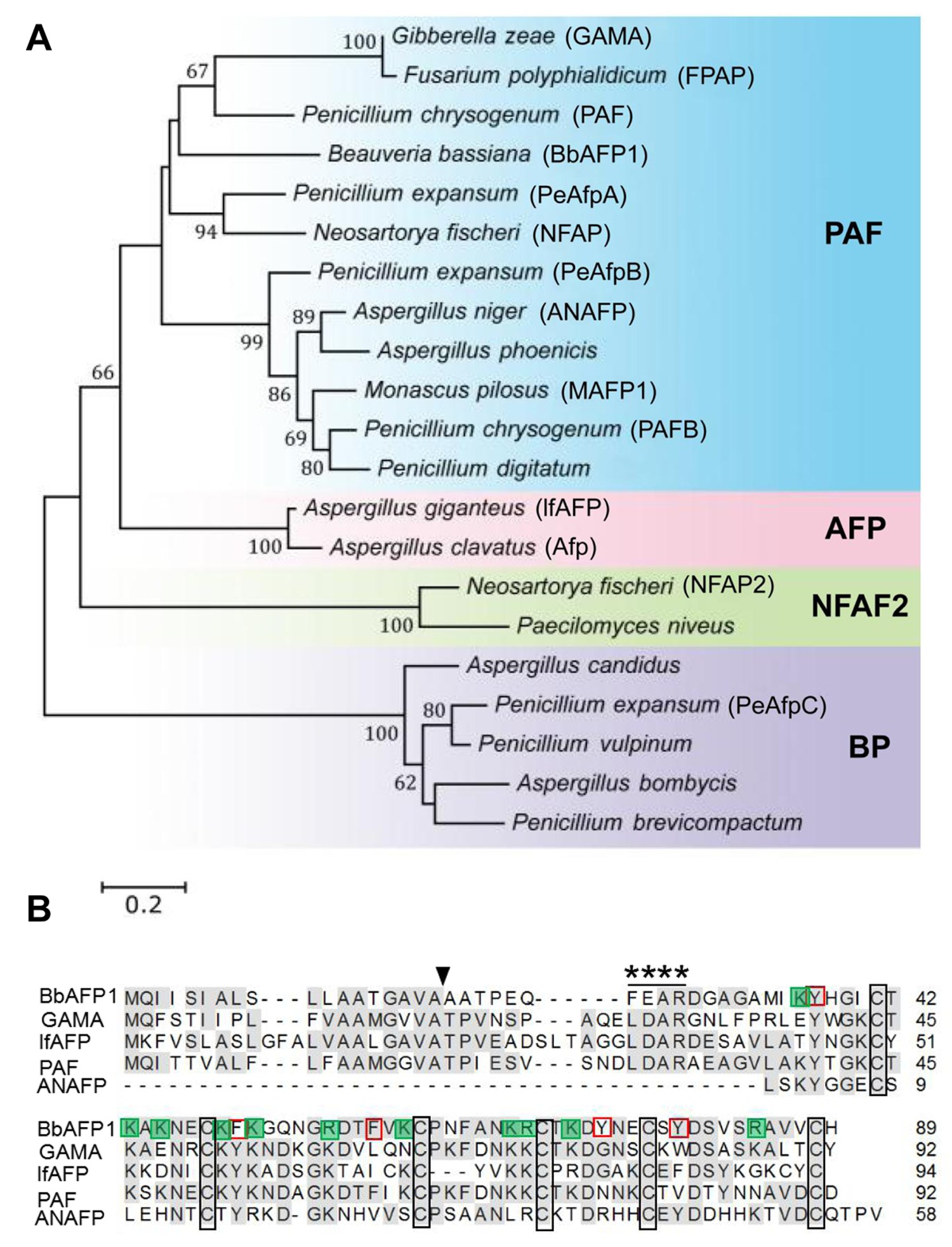

Supplement: S1 Fig — (A) Phylogenetic tree of BbAFP1 and its homologous proteins. Phylogenetic analysis was performed with MEGA soft and as described by Sonderegger et al. (2018). The name of characterized proteins was given in parenthesis. (B) Alignment of protein sequences of BbAFP1, PAF, GAMA and ANAFP by Clustal W method. Identical aa sequences are highlighted in grey, the signal peptide cleavage site is indicated by an arrowhead. The putative prosequence cleavage site of PAF is LDAR (Meyer 2008). A similar site (FEAR) is found in BbAFP1 and marked by four asterisks. In the mature protein region of BbAFP1, aromatic amino acids which are mutated into alanine are indicated with red boxes, Lys and Arg residues are indicated with green fill boxes and conserved Cys residues are indicated with black boxes. (TIF) [file ppat.1008518.s001.tif]

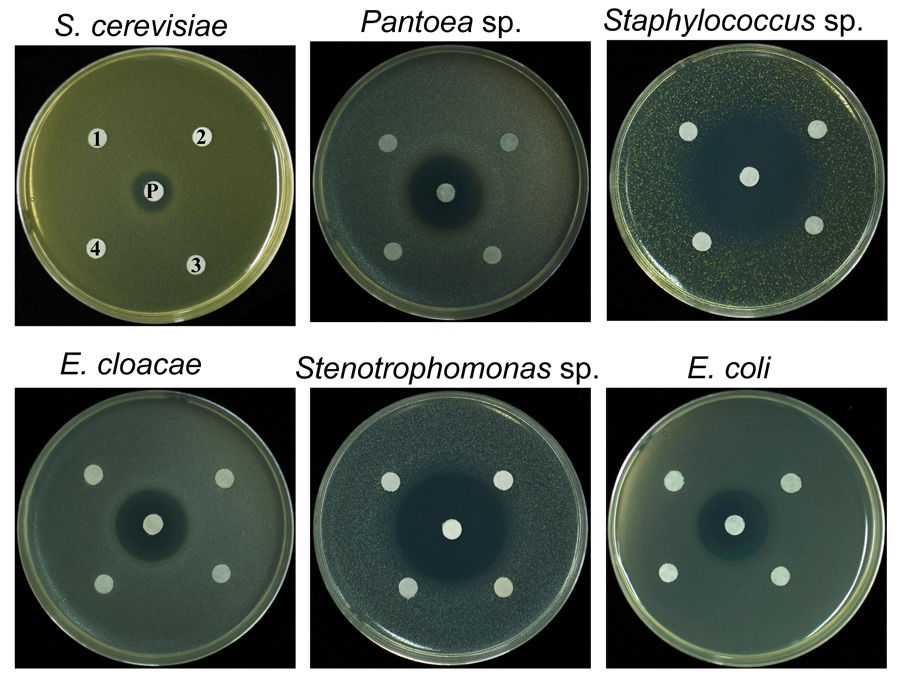

Supplement: S2 Fig — P, indicated positive control (10 μg cefotaxime sodium for bacteria and 50 μg geneticin for yeast); 1–4, indicated 0, 0.5, 1.0 and 2.0 μg BbAFP1 was added, respectively. The same sequence of samples was used in all plates. Bacteria were cultured at 37℃ overnight on LB plates. S. cerevisiae (yeast) was cultured at 30℃ for 2 d on YPDA plate. (TIF) [file ppat.1008518.s002.tif]

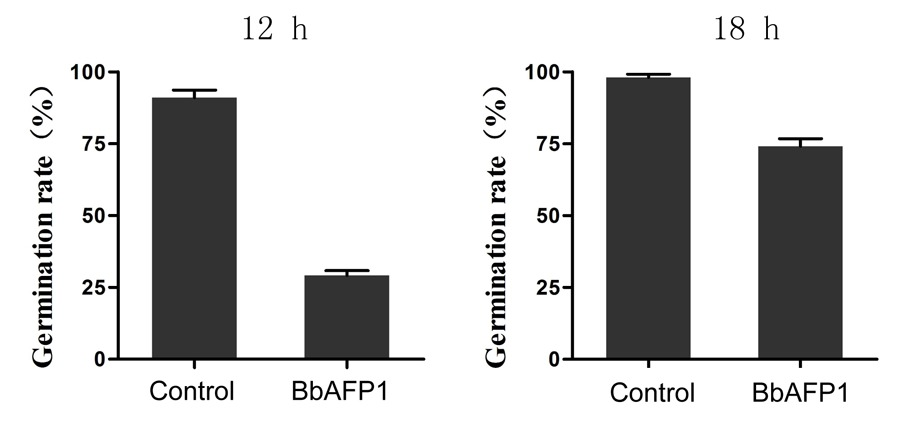

Supplement: S3 Fig — Purified BbAFP1 (5 μM) was added into the conidial suspension of A. brassicae. 20 mM NaAC (pH 5.4) was used as a control. Conidial germination rates of A. brassicae were calculated 12 h or 18 h post-inoculation. All experiments were performed in triplicate with at least three independent biological samples. Error bars = SD. (TIF) [file ppat.1008518.s003.tif]

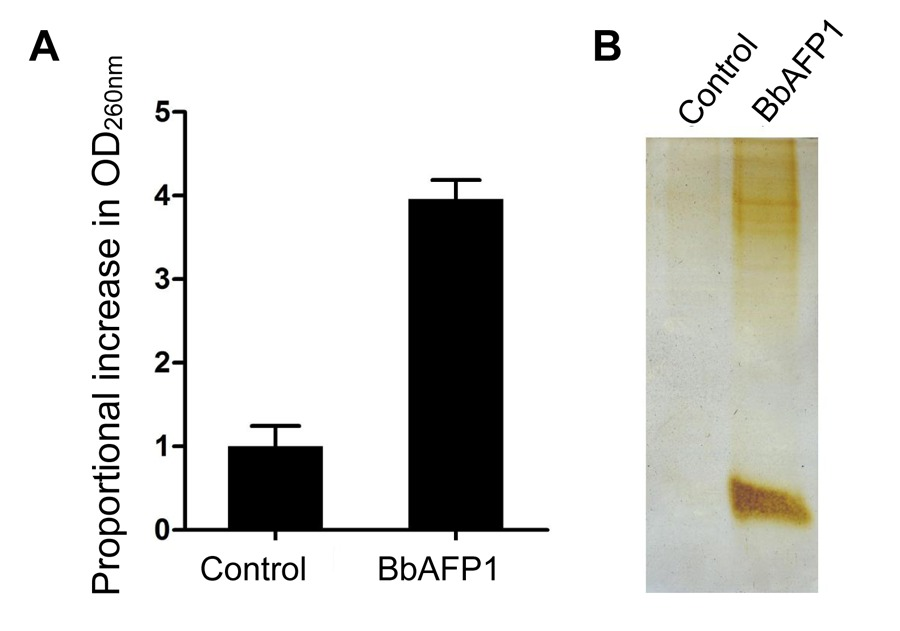

Supplement: S4 Fig — After treated with BbAFP1 (5 μM) for 3 h, the OD260 of DNA/RNA was determined and proteins were run in SDS-PAGE and detected by silver staining. All experiments were performed in triplicate with at least three independent biological samples. Error bars = SD. (TIF) [file ppat.1008518.s004.tif]

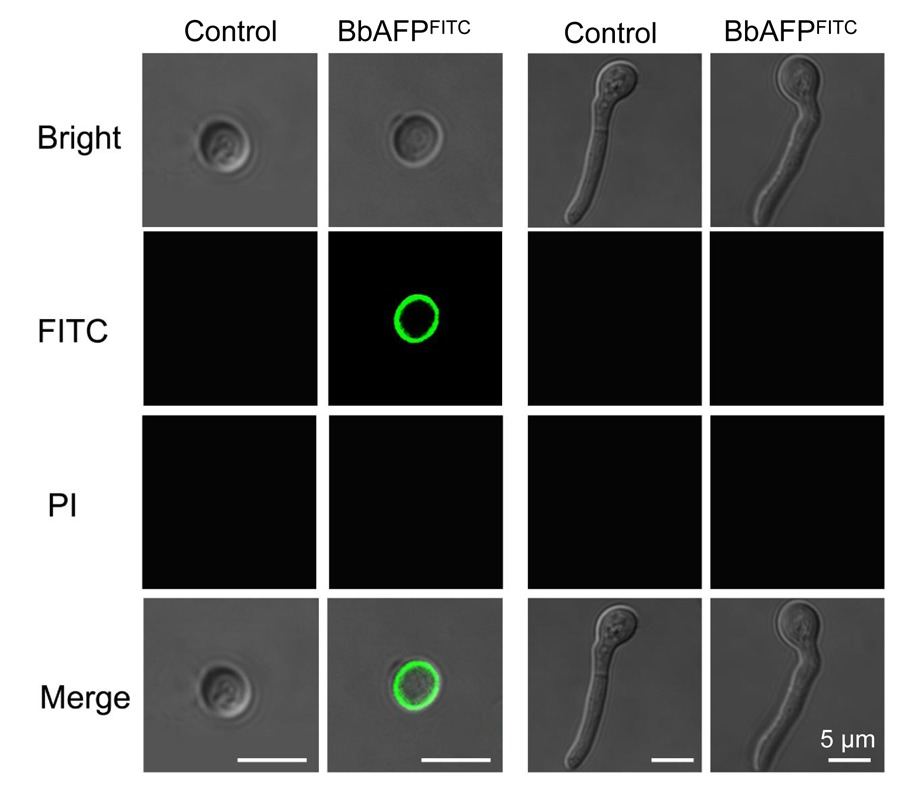

Supplement: S5 Fig — B. bassiana conidia were pretreated with BbAFP1FITC in PDB for 3 h and 15h at 26℃, respectively, then PI was added into the conidia suspension to examine membrane integrity. (TIF) [file ppat.1008518.s005.tif]

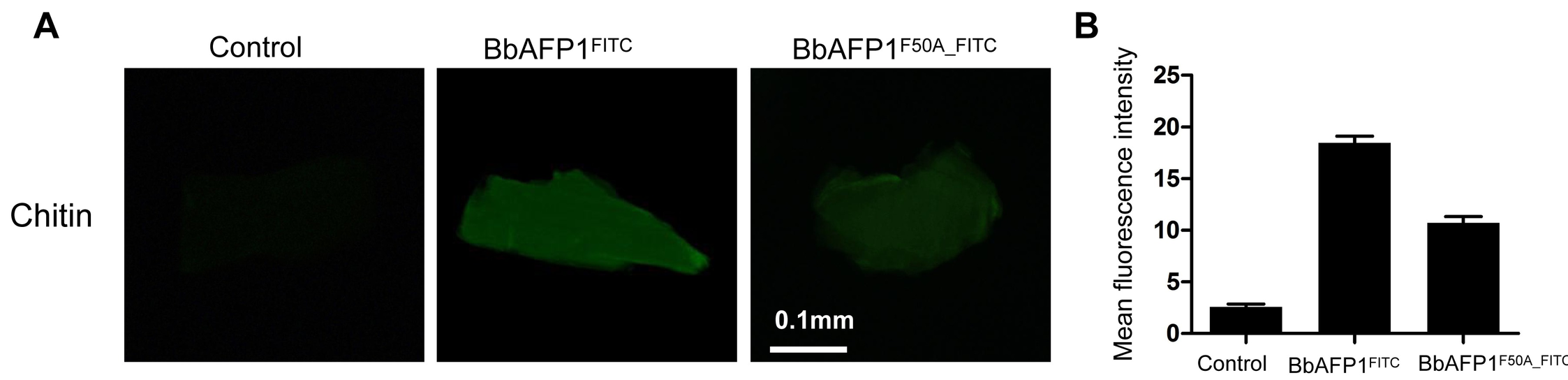

Supplement: S6 Fig — The fluorescence observation (A) and mean fluorescence intensity quantification (B) of FITC on chitin. We quantified the mean fluorescence intensity by ImageJ software and powdered chitin treated with 20 mM potassium phosphate buffer (pH 6.0) was used as a control. Error bars = SD. (TIF) [file ppat.1008518.s006.tif]

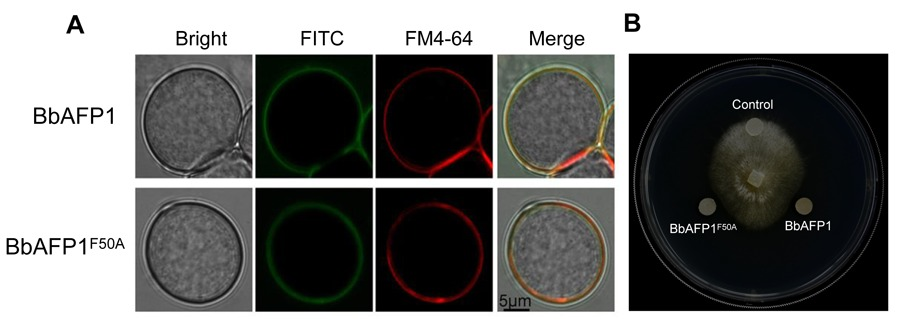

Supplement: S7 Fig — (A) Localization of BbAFP1FITC and BbAFP1F50A_FITC on P. nicotianae conidia. (B) The inhibitory activity of BbAFP1 and BbAFP1F50A against P. nicotianae. BbAFP1/BbAFP1F50A (2 μg) was spotted onto round filter papers near fungal colony. NaAC (20 mM, pH 5.4) was used as a control. (TIF) [file ppat.1008518.s007.tif]

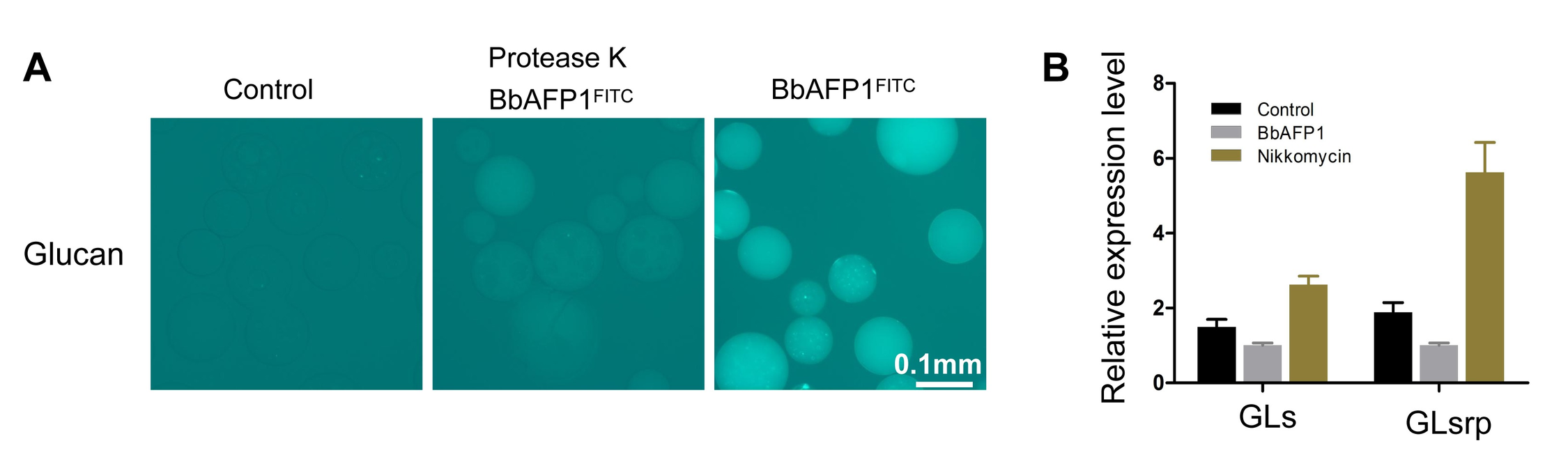

Supplement: S8 Fig — (A) Binding of BbAFP1FITC to glucan was determined as detailed in the Materials and Methods section. Glucan treated with 20 mM potassium phosphate buffer (pH 6.0) and Protease K treated BbAFP1FITC were used as a control. (B) Effects of BbAFP1 on the expression level of glucan synthesis related genes, 1,3-beta-glucan synthase (GLs) and the glucan synthesis regulatory protein gene (GLsrp) in F. graminearum. F. graminearum was treated with BbAFP1 or the chitin synthesis inhibitor nikkomycin for 2 d, after which total RNA was isolated and RT-PCR analysis was performed with β-tubulin as the reference gene as detailed in the Materials and Methods section. All experiments were performed in triplicate. Error bars = SD. (TIF) [file ppat.1008518.s008.tif]

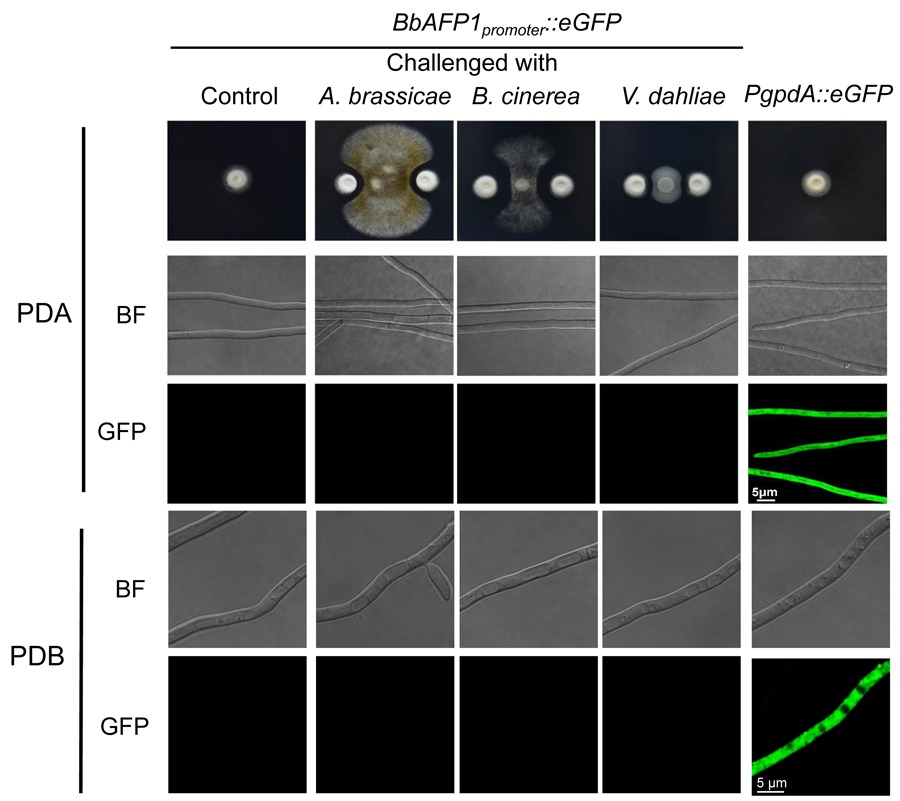

Supplement: S9 Fig — On PDA plates, BbAFP1promoter::eGFP strain was inoculated near the colony edge of several filamentous fungi, including A. brassicae, B. cinerea and V. dahliae (top panel). For liquid medium, BbAFP1promoter::eGFP strain and test fungus were individually pre-cultured in PDB for 2 d, then mixed them together and cultured for additional 24 h. The expression of BbAFP1 was detected by GFP fluorescent observation. (TIF) [file ppat.1008518.s009.tif]

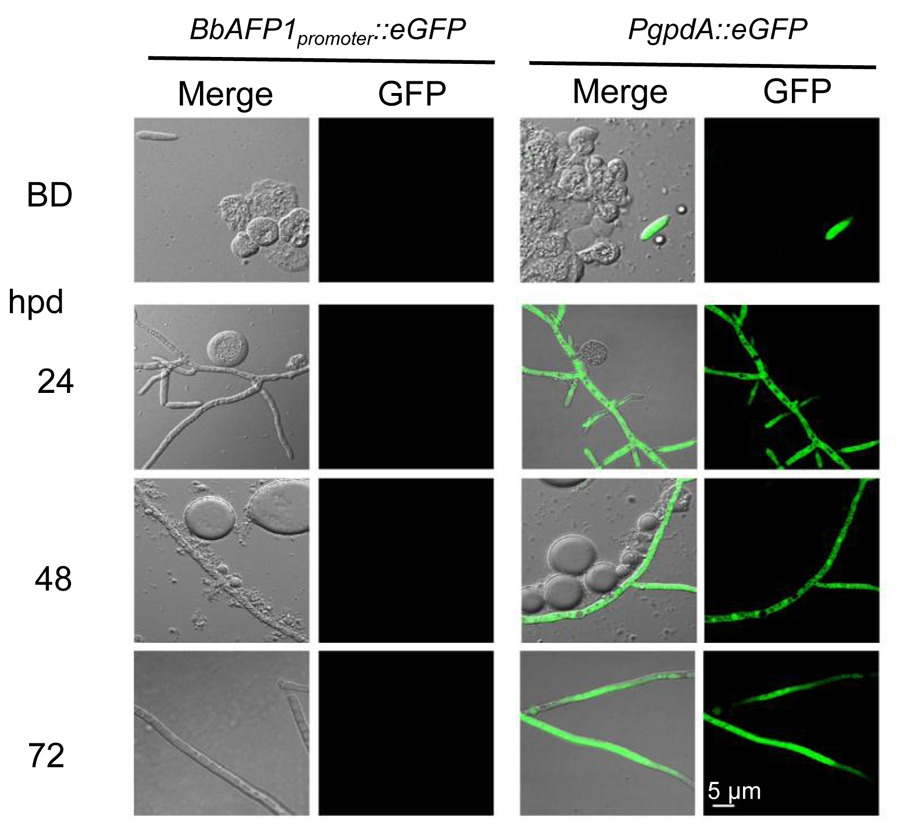

Supplement: S10 Fig — Time course include before G. mellonella death (BD, ~72 h post infection) and 24–72 h post death (hpd). A strain constitutively expressing eGFP (PgpdA::eGFP) was used as positive controls. (TIF) [file ppat.1008518.s010.tif]

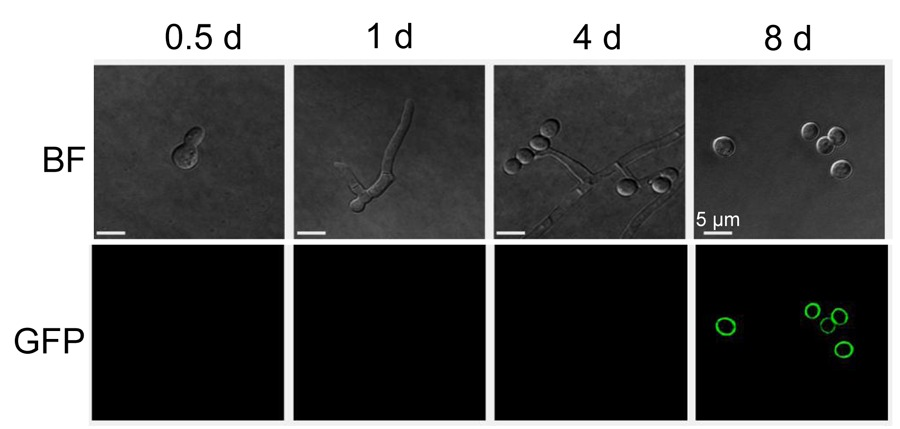

Supplement: S11 Fig — BbAFP1promoter::BbAFP1::eGFP strain was inoculated onto CZA and fluorescent signal was detected during 0.5–8 d. (TIF) [file ppat.1008518.s011.tif]

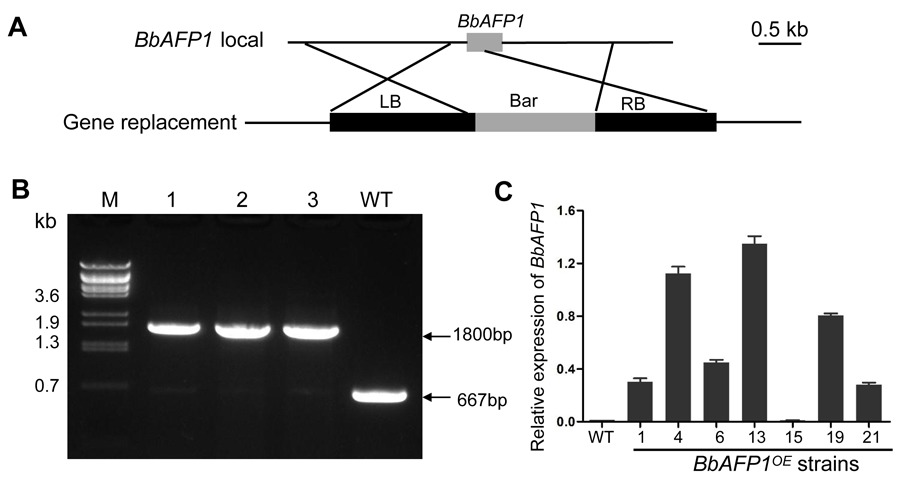

Supplement: S12 Fig — (A) Schematic of construction of ΔBbAFP1 mutants. (B) Screening and confirmation of BbAFP1 knockout strains by PCR. Lane M, Marker 15 (Fermentas), lane 1–3, ΔBbAFP1 mutants, WT, B. bassiana wild type. (C) Screening of BbAFP1 overexpressing strains by real-time PCR. (TIF) [file ppat.1008518.s012.tif]

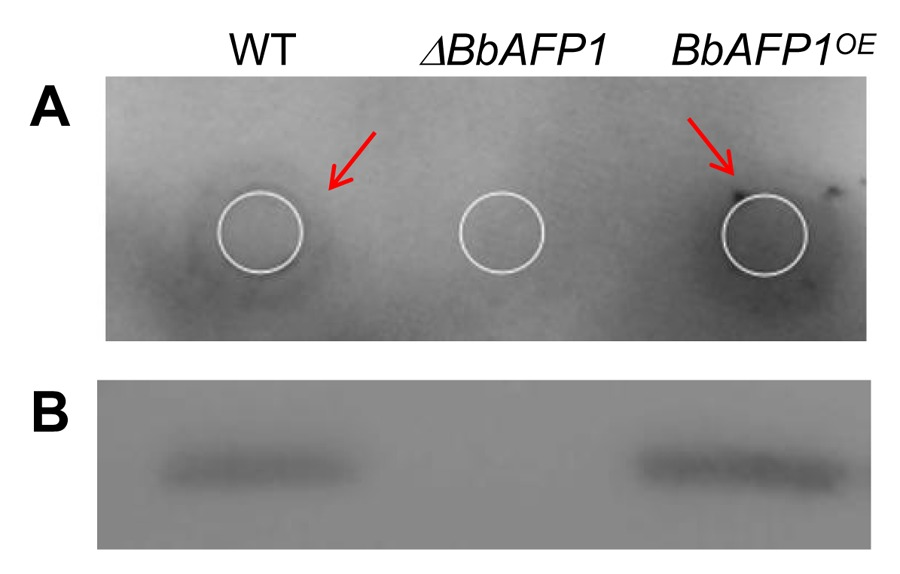

Supplement: S13 Fig — (A) BbAFP1 was detected in situ on agar plates. White circles indicate the inoculation area of B. bassiana conidia. Red arrows indicate the BbAFP1 signal. (B) BbAFP1 was detected in protein extracts from agar. Antibody against BbAFP1 was used. (TIF) [file ppat.1008518.s013.tif]

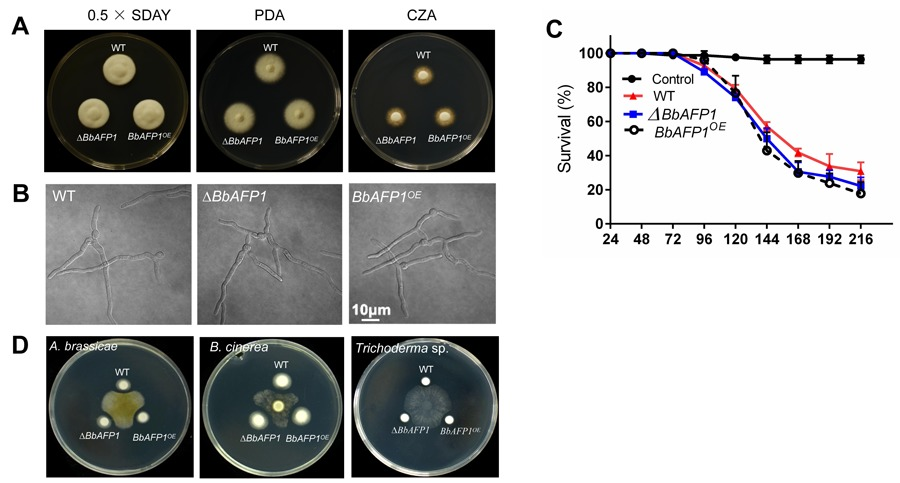

Supplement: S14 Fig — (A) Colony phenotype of various strains. BbAFP1OE, ΔBbAFP1 and B. bassiana wild type strains were inoculated on 0.5 × SDAY, PDA, and CZA plates respectively, and the colony phenotype was observed after cultured the plates at 26 ℃ for 6 days. (B) Hyphal morphology was observed after cultured various stains in PDB for 18 h. (C) Bioassay analysis against G. mellonella larvae. (D) The antagonistic activity of B. bassiana strains against various fungi (the central colony) were analyzed on PDA. (TIF) [file ppat.1008518.s014.tif]

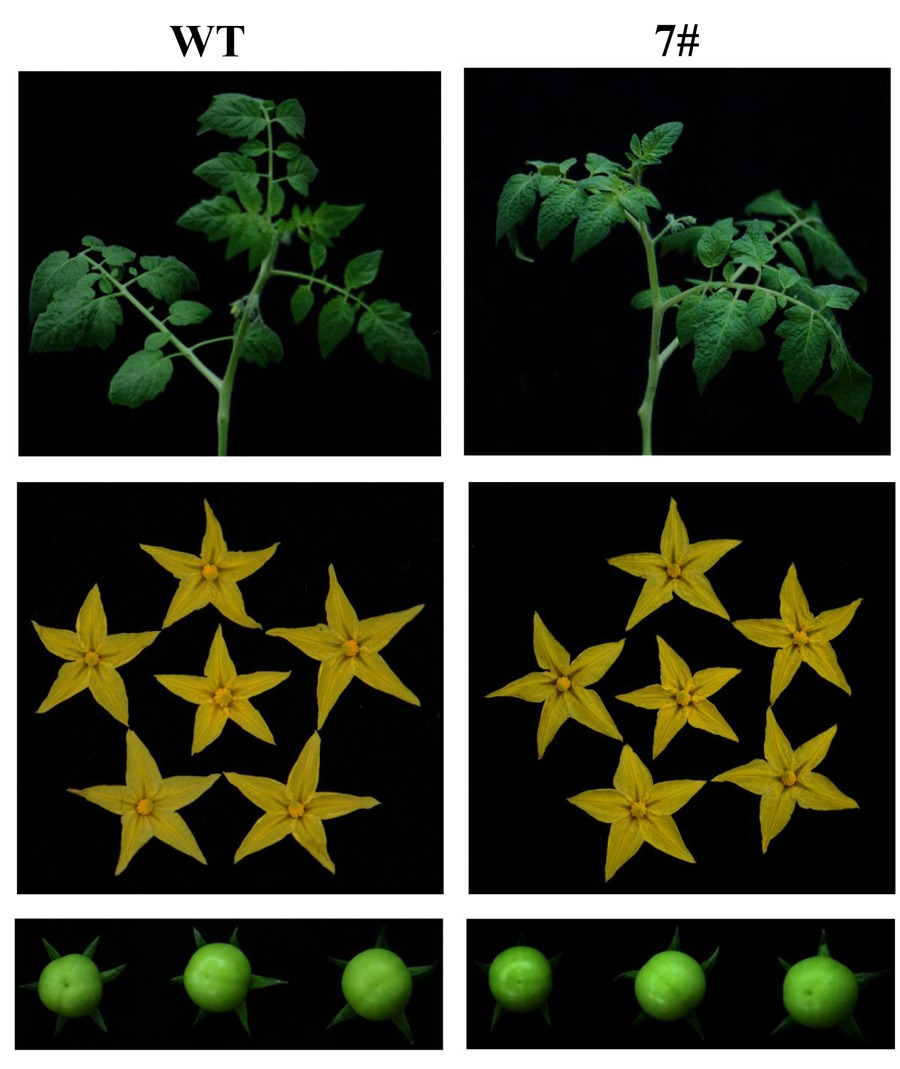

Supplement: S15 Fig — Plant growth, floral development and fruit size were not significantly different between wild-type and BbAFP1 transgenic tomato. WT, wild-type tomato; 7#, transgenic tomato line. (TIF) [file ppat.1008518.s015.tif]
