# Supplementary material for: Characterization of a fungal competition factor: Production of a conidial cell-wall associated antifungal peptide
Source: PLoS Pathog. 2020 Apr 23;16(4):e1008518. doi: 10.1371/journal.ppat.1008518 (PMC7200012; doi:10.1371/journal.ppat.1008518)
Supplement: S1 Table — aPutative parameters. The mature protein of BbAFP1 was deduced by compared the amino acid sequence with that of PAF. The parameters of other fungal AFPs were cited from the references. (DOCX) [file ppat.1008518.s016.docx]

**S1 Table. Parameters of putative BbAFP1 mature protein with several identified fungal AFPs.**

| **Proteins** | **Number of aa** | **Mass (kDa)** | **Cys residues** | **Lys/Arg residues** | **Theoretical pI** | **Reference** |
| --- | --- | --- | --- | --- | --- | --- |
| **^a^BbAFP1** | 61 | 6.8 | 6 | 8/3 | 9.01 | Genebank accession EJP62050.1 |
| **AFP** | 51 | 5.8 | 8 | 12/1 | 9.27 | Wnendt et al. (1994) |
| **lfAFP** | 57 | 6.4 | 8 | 12/1 | 9.05 | Martinez-Ruiz et al. (1997) |
| **ANAFP** | 58 | 6.6 | 6 | 5/3 | 7.14 | Lee et al. (1999) |
| **NAF** | 55 | 6.3 | 6 | 13/0 | 8.93 | Geisen (2000) |
| **PAF** | 55 | 6.3 | 6 | 13/0 | 8.93 | Marx et al. (1995) |
| **^a^GAMA** | 55 | 6.4 | 6 | 12/1 | 9.10 | Genebank accession BK004091 |

^a^Putative parameters.

The mature protein of BbAFP1 was deduced by compared the amino acid sequence with that of PAF. The parameters of other fungal AFPs were cited from the references.
