# Supplementary material for: Characterization of a fungal competition factor: Production of a conidial cell-wall associated antifungal peptide
Source: PLoS Pathog. 2020 Apr 23;16(4):e1008518. doi: 10.1371/journal.ppat.1008518 (PMC7200012; doi:10.1371/journal.ppat.1008518)
Supplement: S2 Table — (DOCX) [file ppat.1008518.s017.docx]

**S2 Table. Primers used in this study.**

| Name | Sequence (5’ to 3’) | Use |
| --- | --- | --- |
| P1  P2  P3  P4  PBbAFPrt-1  PBbAFPrt-2  Pactin-1  Pactin-2  Pgpd-1  Pgpd-2  Pcypa-1  Pcypa-2  Ppafpp1  Ppafpp2  Ppafpp3  Pegfp1  Pegfp2  POEAFP-1  POEAFP-2  PgpdA-1  PgpdA-2  PafpLB1  PafpLB2  PafpRB1  PafpRB2  PBbafpt1  PBbafpt2  Y37A-1  Y37A-2  F50A-1  F50A-2  F59A-1  F59A-2  F65A-1  F65A-2  Y74A-1  Y74A-2  Y79A-1  Y79A-2  PBbAFP-3  PBbAFP-4  PLetubinrt-1  PLetubinrt-2  Pactin-m1  Pactin-m2  PAbreAtr1-1  PAbreAtr1-2  FgTubulrt-1  FgTubulrt-2  FgChs1art-1  FgChs1art-2  FgChs1brt-1  FgChs1brt-2  FgChs2rt-1  FgChs2rt-2  FgChs3rt-1  FgChs3rt-2  FgChs4rt-1  FgChs4rt-2  FgChs6rt-1  FgChs6rt-2  FgChsDrt-1  FgChsDrt-2 | GAATTCACCCCTGAGCAGTTTGAGGC  GCGGCCGCCTAGTGGTGGTGGTGGTGGTGGTGGCACACGACAGCTCGGC  CGGGATCCATGCAGATTATTTCCATCGC  CGGAATTCCTAGTGGCACACGACAGCTC  GAGCAGTTTGAGGCCAGAGA  AGTTGGGGCACTTGACAAAG  GTCAAGTCATCACCATTGGC  GAGGAGCAATGATCTTGACC  GTGTCTTCACCACTACTGAG  TGTAGCCGAGAATGCCCTTG  ATGGCTAACCCCAAGGTCTT  AACTGGGAGCCGTTGGTGTT  GACCTGCAGGCATGCAAGCTTGACGGAATAGATCAATCGTG  TTGCTCACCATGGTGGCGGCTTTGCAAAATTTTGAGGTGATTTT  GCTCACCATGGTGGCGGCACCAGAACCACCACCAGAACCACCGTGGCACACGACAGCTC  GCCGCCACCATGGTGAGCAA  ACGACGGCCAGTGCCAAGCTTTTACTTGTACAGCTCGTCCAT  ACCCTTTTAATCAATAACAAATGCAGATTATTTCCATCGC  GACCTGCAGGCATGCAAGCTTCTAGTGGCACACGACAGCTC  ACGACGGCCAGTGCCAAGCTTTGTTGGGTATGCTCCGGCGC  TTGTTATTGATTAAAAGGGTGA  TATGACCATGATTACGAATTCAGCTAGGTAGCGAGTAAGGC  TCTGTCGACACTAGTGAATTCTCCGTACCTATTTGGGCTTG  GACCTGCAGGCATGCAAGCTTCTTTGTCAAGTGCCCCAACT  ACGACGGCCAGTGCCAAGCTTGCCTGTGACTCCTTTGTTGC  GAGATGATCGGTGATGTTAC  ACATTCGTTGTAGTCCTTGG  GCCCACGGAATTTGCACCAAGGC  CTTGATCATGGCGCCGGCCC  GCCAAGGGCCAGAATGGCAGGGA  CTTGCATTCGTTCTTGGCCT  GCCGTCAAGTGCCCCAACTTTGC  GGTATCCCTGCCATTCTGGC  GCCGCCAACAAGAGATGCACCAA  GTTGGGGCACTTGACAAAGG  GCCAACGAATGTTCATATGACAG  GTCCTTGGTGCATCTCTTGT  GCCGACAGCGTCAGCCGAGCTGT  TGAACATTCGTTGTAGTCCT  cgggatccatgcagattatttccatcgc  cggaattcctagtggcacacgacagctc  ACAGTCTGGTGCTGGTAATA  GAGTGGCAAACCTGGAAT  CACGTTATTCCCGTTGCCGA  ACTCCTTGACGATATCGGGAC  CATCGACTCCGTTCCGCTTA  CGCCACGATGATATCCCACA  ATGGTCGGATTTGCCCCTCTG  TGGTCCTCGATCTCCTTTGTTGA  CTCGCCGCTTTCTATCTCACT  AGCACCTTTTGGTCTGTTTCC  CTTTCAAGGAAGAGGAGGAGGTCG  TGGTGCTGGTAATGCAAACAATG  CGCTTCAGAATGACGAGACCG  CCAACTCCCAACAGAGGATACGAT  CAAGGAAGACAAGGAGGCAGAGC  CACCACGGAACATGCGTACAAC  CCCGGTTGTTTCTGTATGTATCGC  GGAATGTTCGGAGCATCAGAGTTG  GGTCGTTACGGCGCACTAGG  TGAAGACTGTGAGCAGGCTTGATG  CCGCACAAGGTAGTCGCA  CGTCTTTGGTGTCCTTTTAGCC | Heterologous expression  RT-PCR  *B. bassiana* reference genes  BbAFP promoter or BbAFP promoter with BbAFP  eGFP  BbAFP for overexpressing  PgpdA for overexpression  KO strain construction  KO strain screening  Site mutation  Plant expression vector  Tomato reference gene  Reference gene actin for mixture sample  AbreAtr1 of *A. brassicae*  *F. graminearum*  reference genes  *F. graminearum* chitin synthase genes |
| Fggsrprt-1 | GAGGGTGCTACCAACAACGA |  |
| Fggsrprt-2 | CAGTCCTCCAGTTGTCCCAC | *F. graminearum* |
| FgGLsrt-1 | CGACTCGAGGTGGTGTTTCA | glucan synthesis |
| FgGLsrt-2 | CAGAGGAAGCTGAGTGCCAA | related genes |
